# Modifications of the PI3K/Akt/mTOR axis during FeHV-1 infection in permissive cells

**DOI:** 10.3389/fvets.2023.1157350

**Published:** 2023-03-21

**Authors:** Gianmarco Ferrara, Consiglia Longobardi, Sara Damiano, Roberto Ciarcia, Ugo Pagnini, Serena Montagnaro

**Affiliations:** ^1^Department of Veterinary Medicine and Animal Productions, University of Naples Federico II, Naples, Italy; ^2^Department of Mental, Physical Health and Preventive Medicine, University of Campania “Luigi Vanvitelli”, Naples, Italy

**Keywords:** FeHV-1, autophagy, PI3K/Akt/mTOR pathway, autophagy inhibitors, autophagy modulation

## Abstract

FeHV-1 is the causative agent of infectious rhinotracheitis in cats. The relationship between viral infection and the PI3K/Akt/mTOR pathway, as well as its function in crucial physiological processes like as autophagy, apoptosis or the IFN induction cascade is known for other varicelloviruses. However, there is no information on whether autophagy is activated during FeHV-1 infection nor on how this infection modifies PI3K/Akt/mTOR pathway. In this work, we aim to elucidate the involvement of this pathway during cytolytic infection by FeHV-1 in permissive cell lines. Using a phenotypic approach, the expression of proteins involved in the PI3K/Akt/mTOR pathway was examined by Western blot analysis. The findings demonstrated the lack of modifications in relation to viral dose (except for phospho-mTOR), whereas there were changes in the expression of several markers in relation to time as well as a mismatch in the time of activation of this axis. These results suggest that FeHV-1 may interact independently with different autophagic signaling pathways. In addition, we found an early phosphorylation of Akt, approximately 3 h after infection, without a concomitant decrease in constitutive Akt. This result suggests a possible role for this axis in viral entry. In a second phase, the use of early autophagy inhibitors was examined for viral yield, cytotoxic effects, viral glycoprotein expression, and autophagy markers and resulted in inefficient inhibition of viral replication (12 h post-infection for LY294002 and 48 h post-infection for 3-methyladenine). The same markers were examined during Akt knockdown, and we observed no differences in viral replication. This result could be explained by the presence of a protein kinase in the FeHV-1 genome (encoded by the Us3 gene) that can phosphorylate various Akt substrates as an Akt surrogate, as has already been demonstrated in genetically related viruses (HSV-1, PRV, etc.). For the same reasons, the use of LY294002 at the beginning of infection did not affect FeHV-1-mediated Akt phosphorylation. Our findings highlight changes in the PI3K/Akt/mTOR pathway during FeHV-1 infection, although further research is needed to understand the importance of these changes and how they affect cellular processes and viral propagation.

## 1. Introduction

The Phosphatidylinositol 3-Kinase (PI3K)-Protein Kinase B (Akt)-Mammalian Target of Rapamycin (mTOR) axis is a regulatory pathway involved in several biological activities, all related to cell survival and growth (1). PI3K plays an important role because, when activated by cellular or extracellular stimuli, it can initiate various biological processes *via* phosphoryl transfer. This event precedes the recruitment of Akt, a serine/threonine kinase that, when phosphorylated, can stimulate mTOR, another protein kinase that functions as a major autophagy regulator (1).

Viral infection is the most important extracellular stimulus that can activate this pathway (2, 3). Its exploitation optimizes not only viral entry, replication, and assembly but also more complex processes such as latency, reactivation, oncogenic transformation, and modulation of host immune response (4, 5). Indeed, the PI3K/Akt/mTOR axis is a key regulator of many cellular processes (controlling translation, metabolism, and cell death), and can fulfill many viral requirements (5). A variety of regulatory mechanisms for the PI3K/Akt/mTOR axis have been currently described in the literature, which differ by virus and host cell type. Several members of the herpesvirus family are known to interact with this pathway and can benefit from its activation (4). For example, herpes simplex virus (HSV) activates mTOR in infected cells, by producing a Ser/Thr kinase called Us3 instead of using the cellular Akt (6–8). Recent works have already demonstrated that varicella-zoster virus (VZV) requires the phosphorylation of Akt to replicate efficiently (9, 10). Pseudorabies virus (PRV) also induces Akt phosphorylation to inhibit apoptosis of infected cells (11). Human cytomegalovirus (HCMV) indirectly interacts with PI3K/Akt encoding UL38, which directly interacts with tuberous sclerosis protein 2 (TSC2), a protein involved in the suppression of mTOR. K1, a transmembrane glycoprotein encoded by KSHV, interacts with the SH2 domain-containing p85 subunit to activate PI3K/AKT/mTOR signaling (12, 13). A recent study shows that LMP1, the major Epstein-Barr virus (EBV) oncoprotein, interacts with the p85 subunit of PI3K to activate PI3K/Akt signaling (14). Similar mechanisms have been also described in viruses belonging to different families, such as flaviviruses, parvoviruses, coronaviruses, etc., (15–17).

Moreover, modifications of this pathway are involved not only in viral entry and replication but also in the host response process and autophagy machinery, according to previous studies (5, 18).

The relation between Feline Herpesvirus type 1 (FeHV-1), a primary pathogen in cats, and PI3K/Akt/mTOR pathway (as well as the changes that its infection causes in the processes activated by this pathway), has not yet been investigated (19). This work aimed to determine the involvement of this pathway during FeHV-1 cytolytic infection as well as the potential proviral or antiviral role.

## 2. Materials and methods

### 2.1. Cells cultures, viral infections, and chemicals

The first experiment was designed to demonstrate how viral dose affects the PI3K/Akt/mTOR activation caused by FeHV-1. Briefly, Crandell-Rees Feline Kidney Cells (CRFK) with 90% confluency, cultured using Dulbecco's Modified Eagle's Medium (DMEM; Corning) containing 10 % fetal bovine serum (FBS) and 1% antibiotics (Penicillin-Streptomycin Solution, Corning), were infected using different multiplicities of infection (MOI) of FeHV-1 strain Ba/91 (MOI 0.1; 1; 10), kindly provided by Prof. C. Buonavoglia, School of Veterinary Medicine of Bari. Infected and control cells were washed twice with phosphate-buffered saline (PBS) after 2 h of viral adsorption, and then incubated in fresh DMEM for 24 h. In addition to control and infected cells a UV-inactivated FeHV-1 was used to determine whether the replication-incompetent FeHV-1 could also activate the pathway. In order to assess the time dependence of FeHV-1-induced PI3K/Akt/mTOR activation we infected CRFK monolayers using a MOI 1 at different time points (3-6-12-24-48-72 hours post infection). Pellets were collected and used for western blot analysis.

In another set of experiments, we examined the proviral or antiviral relationship between the PI3K/Akt/mTOR axis and FeHV-1. 1 h before infection, CRFK monolayers were incubated with two PI3K-inhibitors: 50 μM LY294002 (Cell Signaling) and 10 mM 3-methyladenine (3-MA) (Sigma) (9, 15). Cells were incubated for the indicated times (12, 24, 48, 72 h). We collected pellets, supernatants, and cryolysates from both control and infected cells, and employed them for the subsequent analysis. The same experiment was performed using 50 nM rapamycin (RAP) (Sigma), a specific activator of mTOR (three hours before infection) (20). The effects of LY294002 were further investigated to assess whether changes in Akt phosphorylation occurred at the earliest stages of infection (15, 30, and 60 min).

### 2.2. 3-(4,5-dmethylthiazol-2-yl)-2,5-diphenyl tetrazolium bromide (MTT) assay

The previously described experiments were replicated in 96-well plates with the aim to assess the cell viability using 3-(4,5-dmethylthiazol-2-yl)-2,5-diphenyl tetrazolium bromide (MTT; SERVA). In a summary, each well received 100 μl of the reagent (0.5 mg/mL) and was maintained at 37°C. Addition of 100 μl of the solubilization buffer (dimethylsulfoxide) after 1 h allowed dissolution of formazan crystals from mitochondria (21). A spectrophotometer was used to measure optical absorbance at 570 nm after of incubation at 37°C for 3 h. This assay was also used to evaluate the cytotoxic effects of AKT silencing. Each experiment was carried out in triplicate. Results were determined using the control group as a reference and expressed it as a percentage of cell viability.

### 2.3. Western blot analysis

Cultured CRFK from each experiment were washed with 1X PBS, scraped, and centrifugated. The pellets were then lysed for 30 min at 4°C in an appropriate lysis buffer (1 mM EDTA, 150 mM NaCl, 50 mM TRIS-HCL pH7.5). Protease and phosphatase inhibitors (Sigma) were added to each sample. The protein concentration of each sample was established using the Bradford assay (Biorad). The following electrophoresis run was performed with acrylamide gels (Biorad) transferred into a polyvinylidene difluoride (PVDF) or nitrocellulose (Biorad) membrane using a TransBlot Turbo (Bio-Rad). After 1 hof incubation with blocking solution (5% Bovine Serum Albumin; Serva), the membranes were incubated overnight with a panel of specific monoclonal antibodies: monoclonal antibody against FeHV-1 (Novus biological), Beclin-1 (Cell Signaling), β-Actin (Santacruz), Akt (Cell Signaling), p-Akt 1/2/3 (Santacruz), PI3K class III (Cell Signaling), mTOR (Cell Signaling), and pospho-mTOR (Cell Signaling). Incubation with specific secondary peroxidase-conjugated antibodies (Cell Signaling) for 1 h, preceded visualization with ChemiDoc Blot Scanner (Bio-Rad). The levels of protein expression were assessed by densitometric analysis using Image Lab software.

### 2.4. Determination of viral titers

Supernatants from previous experiments were serially diluted and employed to infect CRKF cultured in 96-well plates with DMEM supplemented with 2% FBS. The Reed-Muench method was used to determine the virus titer. Using a commercial kit (Qiagen), DNA was isolated from aliquots of the same supernatants, measured, and used as a template for gene expression analysis. A SYBR-green real-time PCR amplifying the thymidine kinase (TK) gene (forward primer: 5' TGTCCGCATTTACATAGATGG 3'; reverse primer: 5'GGGGTGTTCCTCACATACAA 3'), was carried out using the CFX 96 Touch real-time PCR detection system (22). Gene expression was measured using a standard curve generated with crude viral DNA (TCID _50_ 10^7^) in serial dilutions.

### 2.5. RNA interference of Akt-1

To determine the effects of cell autophagy on viral replication, a small interfering RNA (siRNA) approach was chosen. The sequence of siRNA targeting AKT-1 (sense 5' GCGUGACCAUGAACGAGUUtt 3' and antisense 5'AACUCGUUCAUGGUCACGCgg 3') was used to transfect CRFK permissive cells using Lipofectamine 3000 (Thermo Fisher) according to the manufacturer's instructions. After 24 h, monolayers were infected with FeHV-1 using a MOI of 1. At 24 h post infection (p.i.), supernatants and pellets were processed as described above. The siRNA efficiency was assessed by measuring protein expression in a Western blot analysis using a specific Akt antibody (Santacruz) (23).

### 2.6. Statistical analysis

Three independent replicates were performed for each experiment. One-way ANOVA and multiple *t*-tests were used to analyze the statistical significance of the variables (GraphPad Prism 6.0), which are expressed as the mean standard deviation (SD). A *P*-value < 0.05 was considered significant.

## 3. Results

### 3.1. PI3K/Akt/mTOR modifications during FeHV-1 infection as a function of viral dose and time

**Figure 1:**
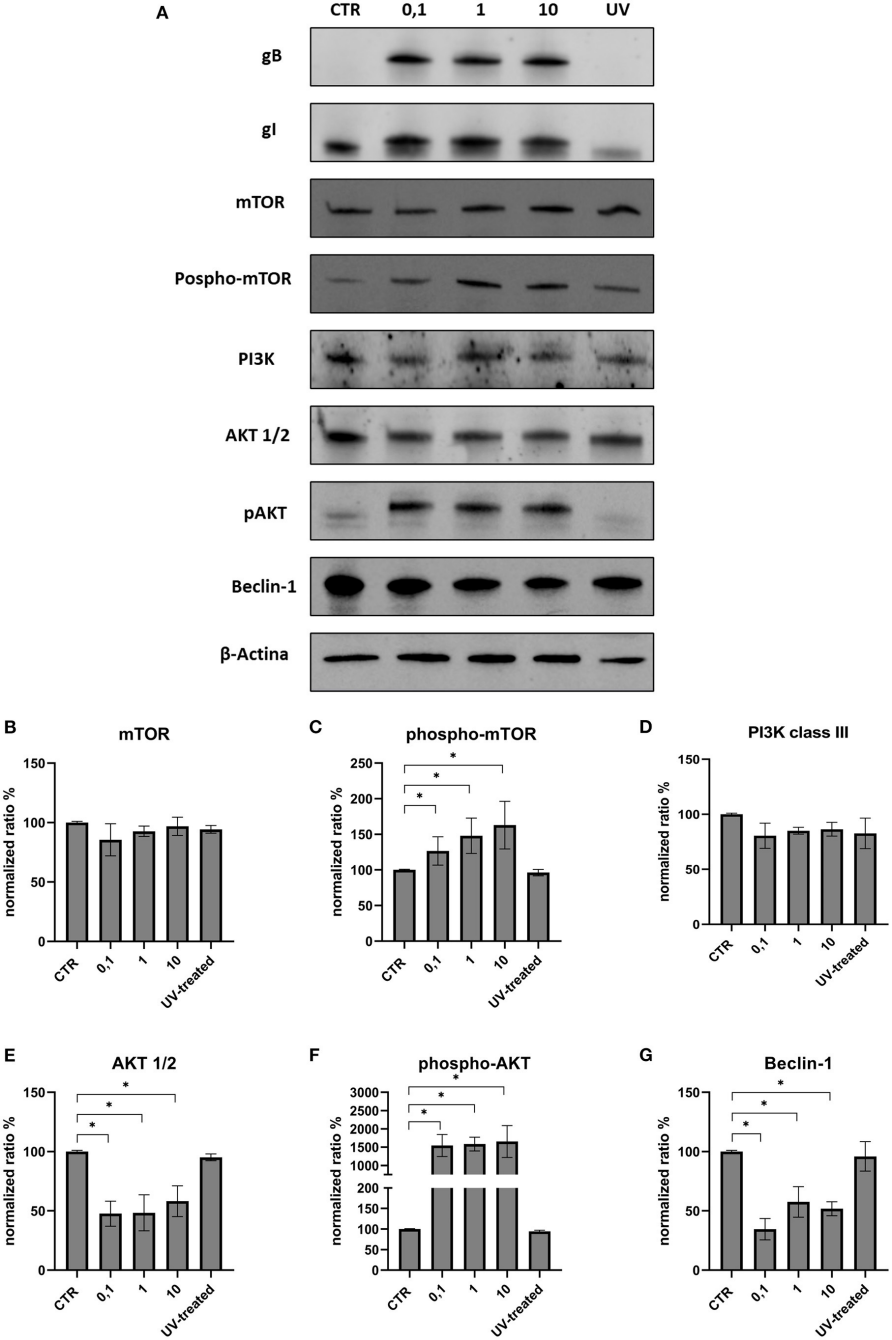
Modification of the PI3K/AKT/mTOR axis according to viral dose. **(A)** Western blot: differences in the expression of several autophagy markers between control, infected cells (MOI 0.1, 1 and 10) and UV-inactivated virus. **(B)** Intensity of protein bands: mTOR. **(C)** Intensity of protein bands: phospho-mTOR. **(D)** Intensity of protein bands: PI3K class III. **(E)** Intensity of protein bands: Akt 1/2. **(F)** Intensity of protein bands: phospho-AKT. **(G)** Intensity of protein bands: Beclin-1. A single representative actin has been inserted into the figure. Individual actins from each membrane, as well as full-size membranes, are available in the Supplementary material. Results were expressed as means ±SD from three independent experiments (*P < 0.05).

**Figure 2:**
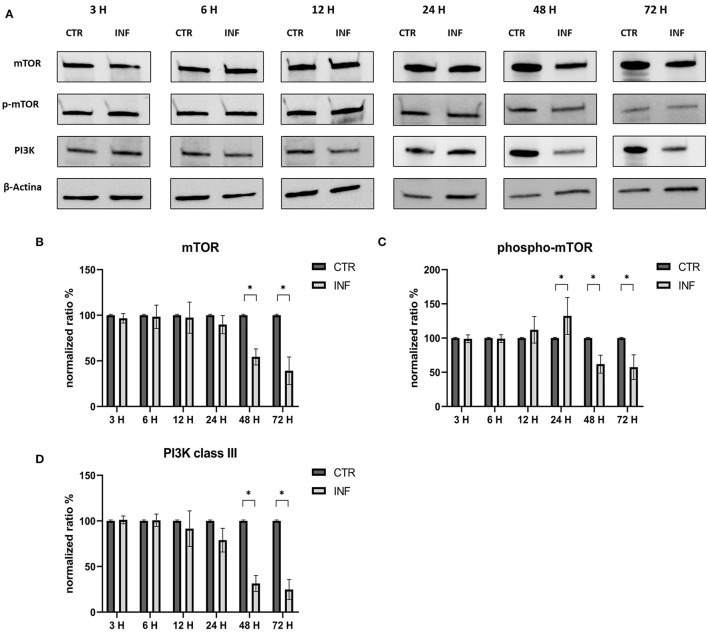
Modification of the PI3K/AKT/mTOR axis (mTOR, Phospho-mTOR and PI3K) according to time of infection. **(A)** Western blot: differences in the expression of several autophagy markers (mTOR, phospho-mTOR and PI3K) between control and infected cells at 3, 6, 12, 24, 48, 72 h post infection. **(B)** Intensity of protein bands: Akt 1/2. **(C)** Intensity of protein bands: phospho-Akt. **(D)** Intensity of protein bands: Beclin-1. A single representative actin has been inserted into the figure. Individual actins from each membrane, as well as full-size membranes, are available in the Supplementary material. Results were expressed as means ±SD from three independent experiments (*P < 0.05).

The effects on the PI3K/Akt/mTOR axis (PI3K, Akt, phospho-Akt, mTOR, and phospho-mTOR) during FeHV-1 infection were examined in Western blot analysis. We found an increase in Akt phosphorylation in infected cells and a decrease of Beclin-1, but this was consistent across different MOI (Figure 1). Viral dose was not associated with significant changes in PI3K or mTOR expression. Pospho-mTOR was the only marker that showed a progressive increase in expression. We found no change in UV-treated viral cells. In subsequent experiments, we used a MOI of 1 at various times. Starting 24 h post infection (Figure 2), we found a simultaneous and significant decrease in PI3K and an increase phospho-mTOR (that subsequently decreased at 48 and 72 h p.i.). Expression of mTOR did not decrease until 48 hours post infection. Beclin-1 expression began to decrease 24 h after infection and continued to decline over 72 h. The phosphorylation of Akt was a surprising finding because it was phosphorylated in infected cells 3 h post infection (Figure 2), when no other markers had changed. Furthermore, constitutive Akt was decreased only 12 h after infection. This impairment of PI3K/Akt/mTOR pathway activation suggests that the virus can interact with the above autophagy markers and activate them independently.

### 3.2. Effects of induction and inhibition of PI3K/Akt/mTOR during FeHV-1 infection

**Figure 3:**
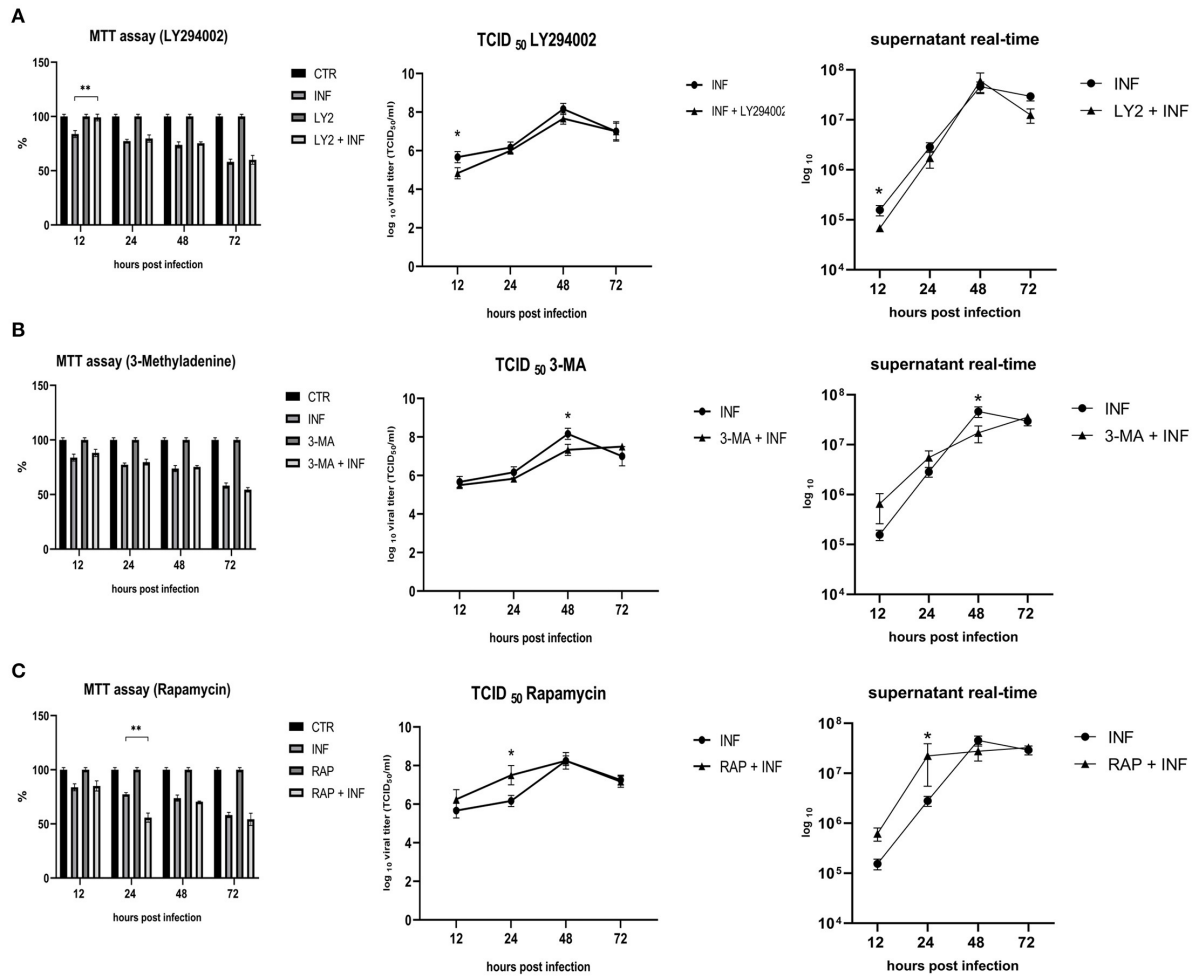
Effects of LY294002, 3-Methyladenine and rapamycin on cell viability and viral proliferation. **(A)** Effects of LY294002 (LY). **(B)** Effects of 3-Methyladenine (3-MA). **(C)** Effects of Rapamycin (RAP). MTT assay results were expressed as means ±SD from three independent experiments (**P < 0.05). TCID50 and real-time PCR results were expressed in a logaritimic scale as means ±SD from three independent experiments (*P < 0.05).

**Figure 4:**
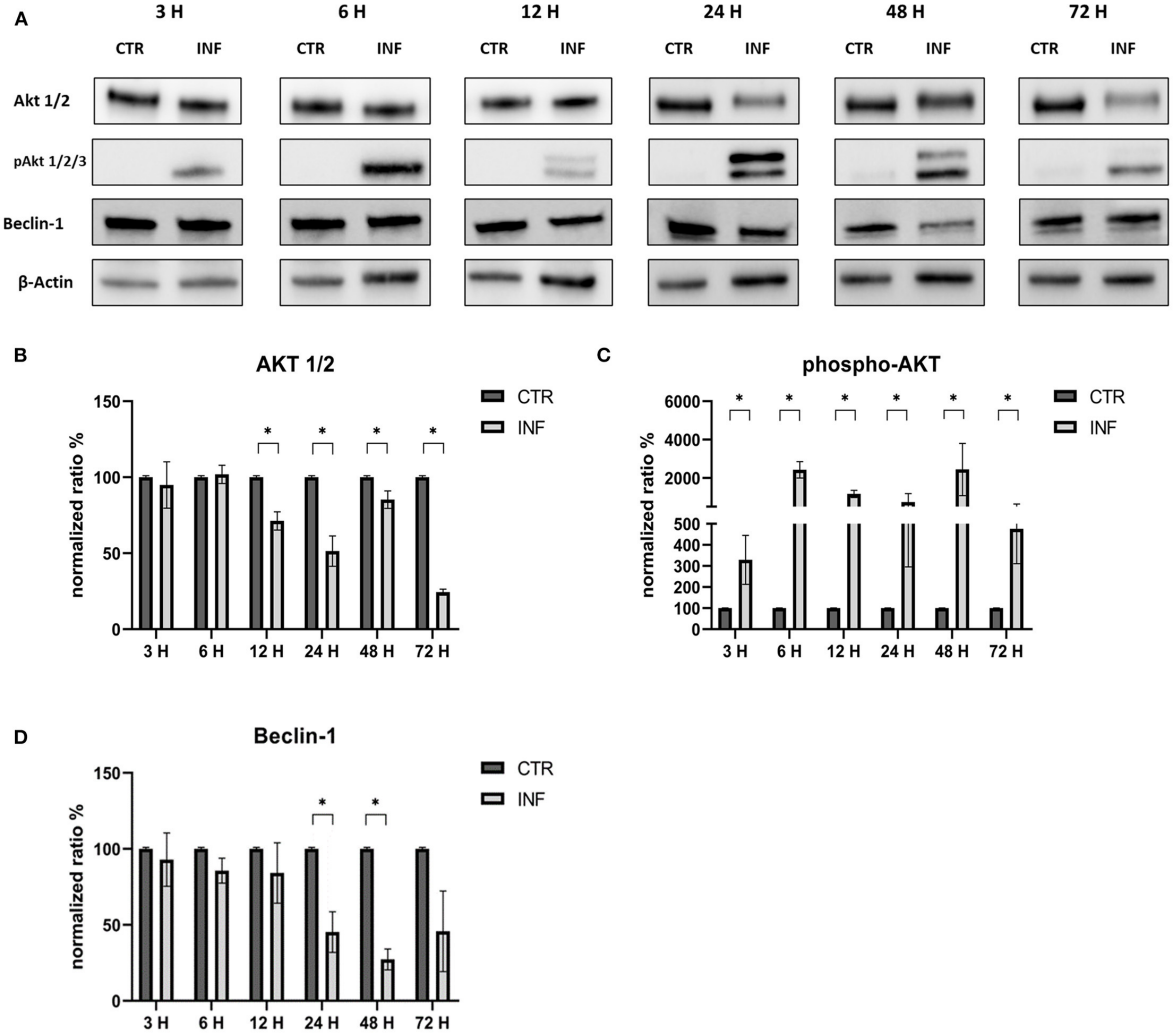
Modification of the PI3K/AKT/mTOR axis (Akt, phospho-Akt, and Beclin-1) according to time of infection. **(A)** Western blot: differences in the expression of several autophagy markers (Akt, phospho-Akt and Beclin-1) between control and infected cells at 3, 6, 12, 24, 48, 72 h post infection. **(B)** Intensity of protein bands: Akt 1/2. **(C)** Intensity of protein bands: phospho-Akt. **(D)** Intensity of protein bands: Beclin-1. A single representative actin has been inserted into the figure. Individual actins from each membrane, as well as full-size membranes, are available in the Supplementary material. Results were expressed as means ±SD from three independent experiments (*P < 0.05).

Permissive CRFK cells were treated with autophagy inducers and inhibitors before infection with FeHV-1 to determine the effects on viral proliferation (viability, viral titers using TCID_50_ and real-time PCR). LY294002 and 3-MA, which inhibit Akt and phosphatidylinositol 3-kinase pathway, were used as early-stage autophagy inhibitors. RAP was used as an autophagy inducer to decrease mTOR activity and promote cell autophagy. MTT assay was used to compare the viability of treated and infected cells (Figure 3). After 12 h, we found that LY294002 significantly increased viability compared with the infected group. FeHV-1-mediated cytotoxicity on permissive cells increased with the use of RAP, resulting in significantly lower viability values at 24 h post infection (Figure 4). No significant modifications were observed when 3-MA was used in association with FeHV-1 infection. CRFK cell viability was not significantly affected when autophagy was altered using the inhibitors and inducers described.

**Figure 5:**
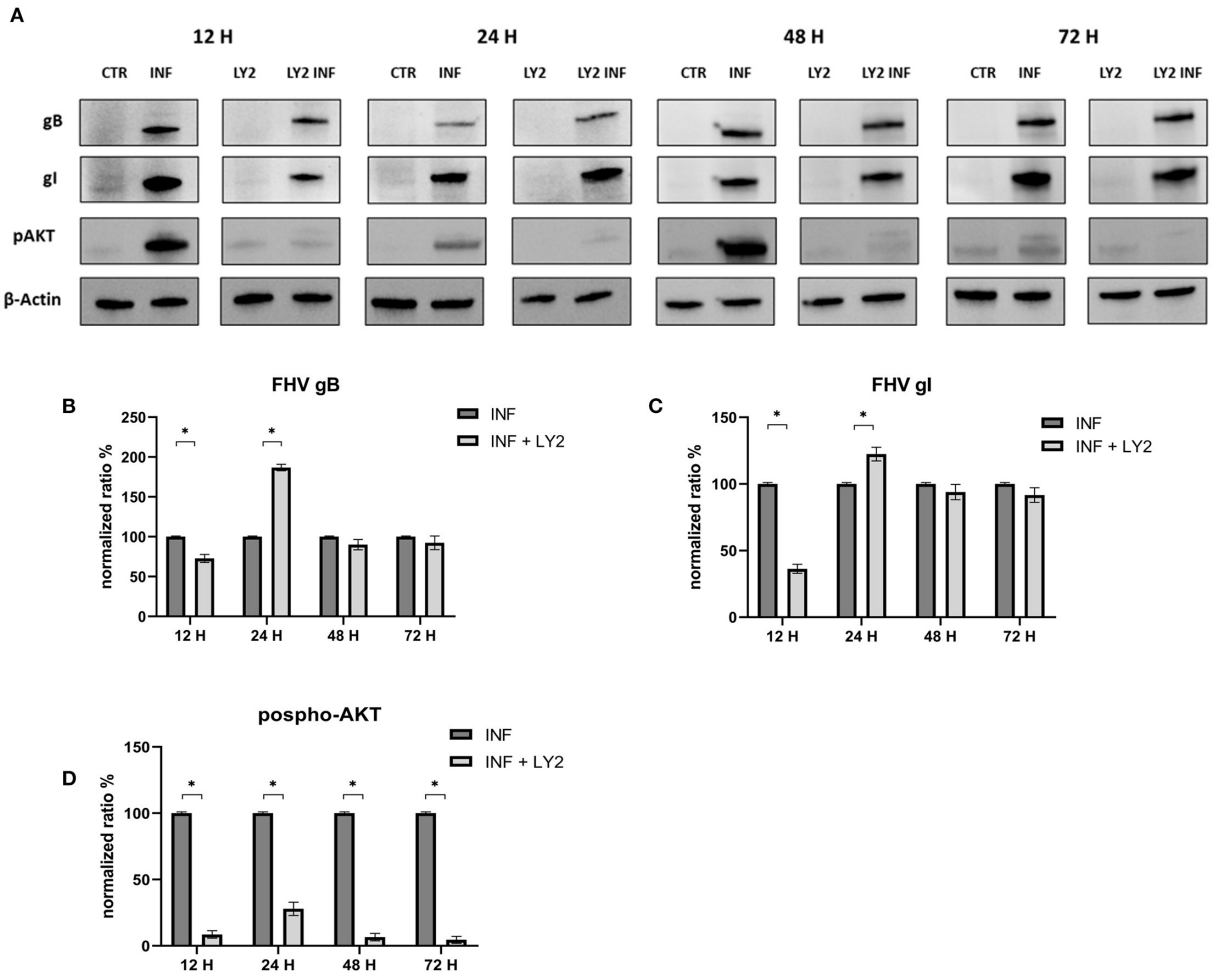
Chemical inhibition of the PI3K/AKT/mTOR axis with LY294002. **(A)** Western blot: Differences in p-AKT, gB and gI expression between control and infected cells in the presence or absence of LY294002. **(B)** Intensity of protein bands: gB. **(C)** Intensity of protein bands: gI. **(D)** Intensity of proteins bands: phospho-Akt. A single representative actin has been inserted into the figure. Individual actins from each membrane, as well as full-size membranes, are available in the Supplementary material. Results were expressed as means ±SD from three independent experiments (*P < 0.05).

**Figure 6:**
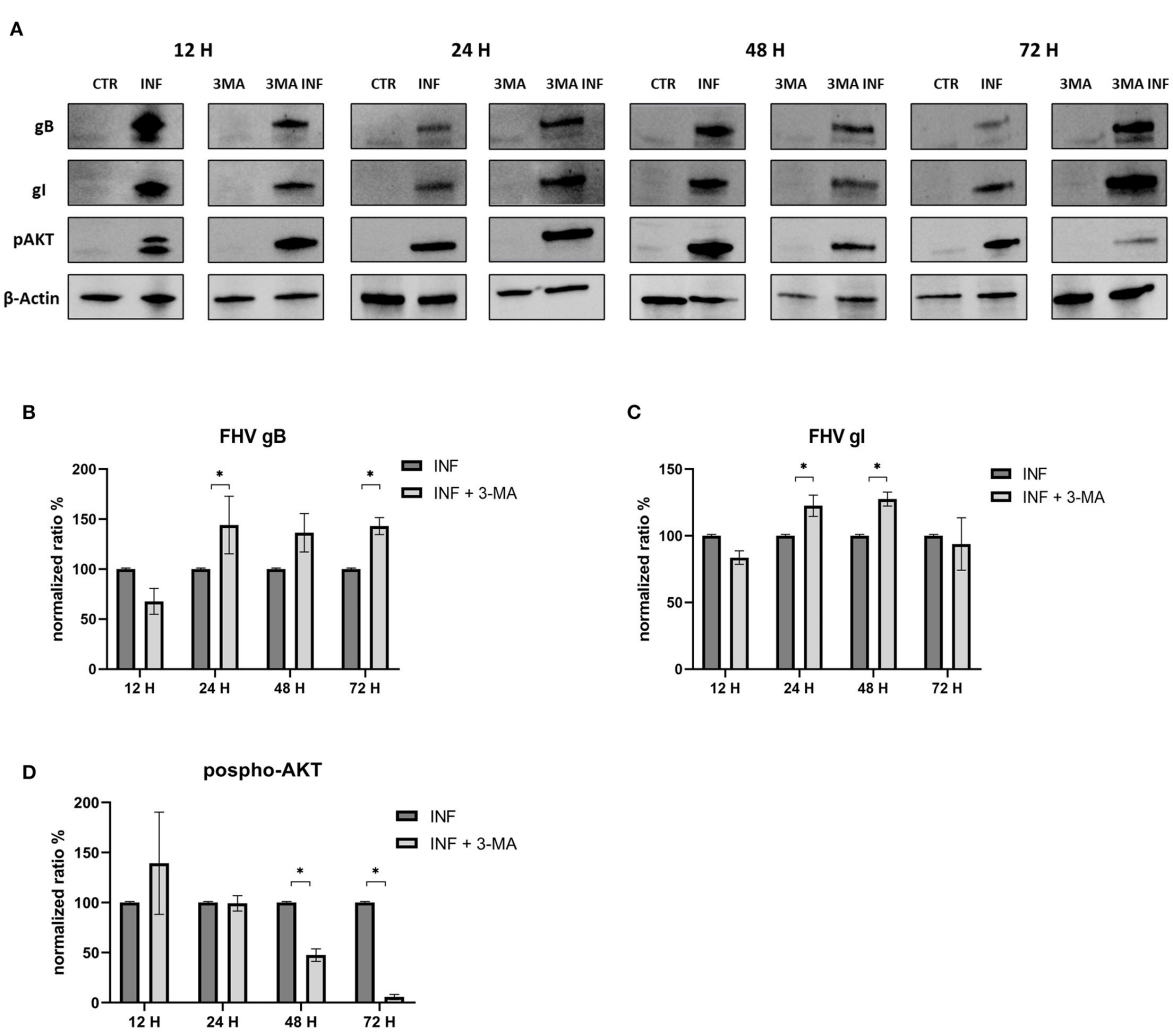
Chemical inhibition of the PI3K/AKT/mTOR axis with 3-Methyladenine. **(A)** Western blot: Differences in p-AKT, gB and gI expression between control and infected cells in the presence or absence of 3-Methyladenine. **(B)** Intensity of protein bands: gB. **(C)** Intensity of protein bands: gI. **(D)** Intensity of proteins bands: phospho-Akt. A single representative actin has been inserted into the figure. Individual actins from each membrane, as well as full-size membranes, are available in the Supplementary material. Results were expressed as means ±SD from three independent experiments (*P < 0.05).

Furthermore, we evaluated how the aforementioned inducers and inhibitors affected viral titers. While the use of LY294002 significantly reduced viral titers in the first 12 h, the use of 3-MA had no tangible impact on the virus titers (we observed a significant decrease in viral titers only at 48 h post infection). RAP treatment enhanced the virus titer 24 h after infection. Furthermore, p-AKT levels were evaluated for LY294002 and 3-MA, and phospho-mTOR/mTOR levels were assessed for RAP. The use of LY294002 resulted in a significant reduction of Akt phosphorylation (12 h p.i.) (Figure 5). An intriguing result was observed for gB and gI expressions that appeared to be transiently reduced 12 h p.i. This phenomenon may be attributable to the impediment in viral entry caused by inhibition of Akt phosphorylation (at least for what concerns cellular Akt). After this time, the levels of these glycoproteins increased (24 h p.i.) and returned to the levels observed in the untreated group. Treatment with 3-MA resulted in changes in and Akt phosphorylation (Figure 6), even if less effectively than LY294002. The expression of viral glycoproteins increased rather than decreased at various times (**Figure 9**). A reduction was observed at 48 h post infection.

**Figure 7:**
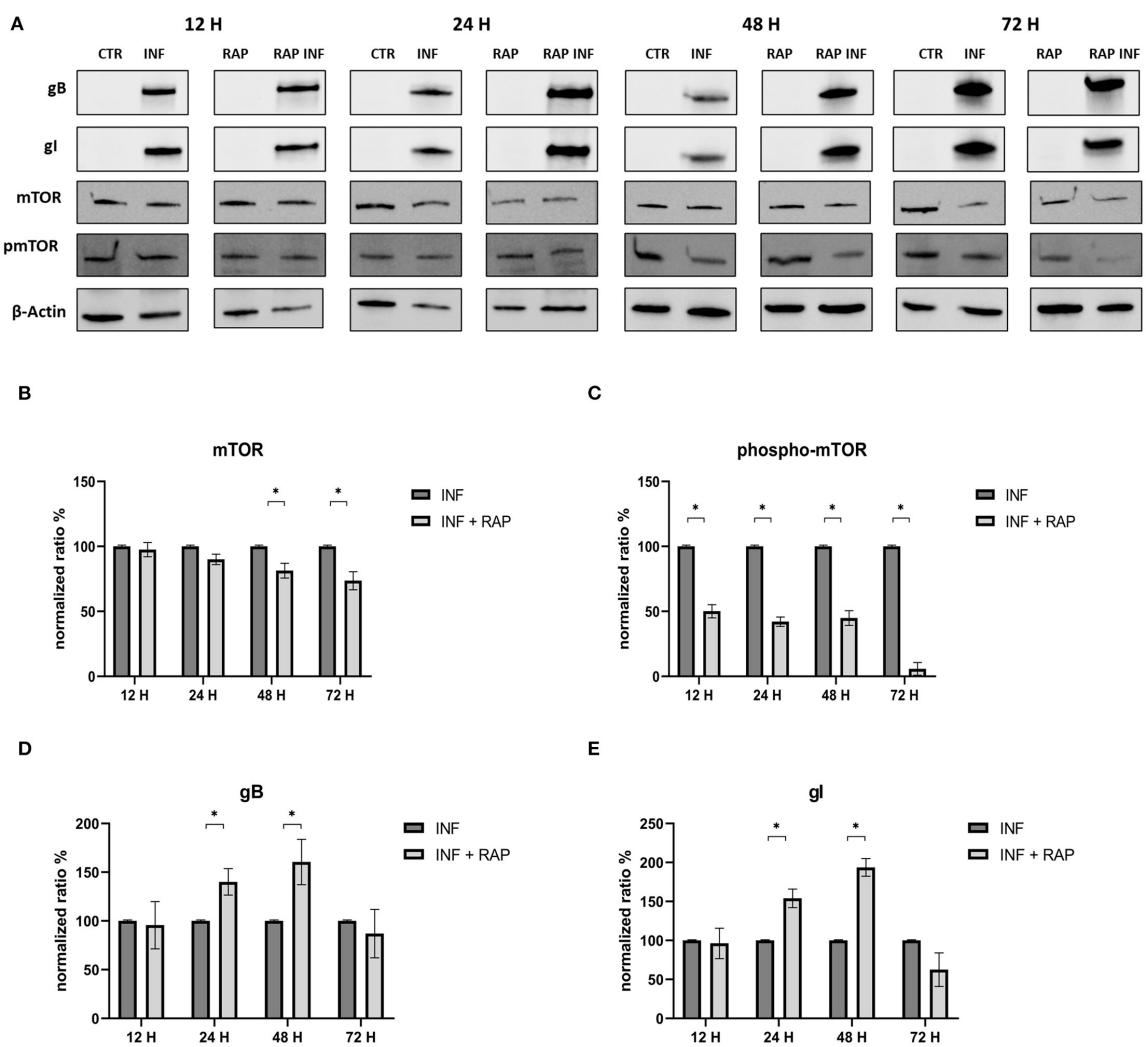
Chemical induction of the PI3K/AKT/mTOR axis with Rapamycin. **(A)** Western blot: Differences in mTOR, phospho-mTOR, gB and gI expression between control and infected cells in the presence or absence of Rapamycin. **(B)** Intensity of protein bands: mTOR. **(C)** Intensity of protein bands: phospho-mTOR. **(D)** Intensity of proteins bands: gB. **(E)** Intensity of protein bands: gI. A single representative actin has been inserted into the figure. Individual actins from each membrane, as well as full-size membranes, are available in the Supplementary material. Results were expressed as means ±SD from three independent experiments (*P < 0.05).

**Figure 8:**
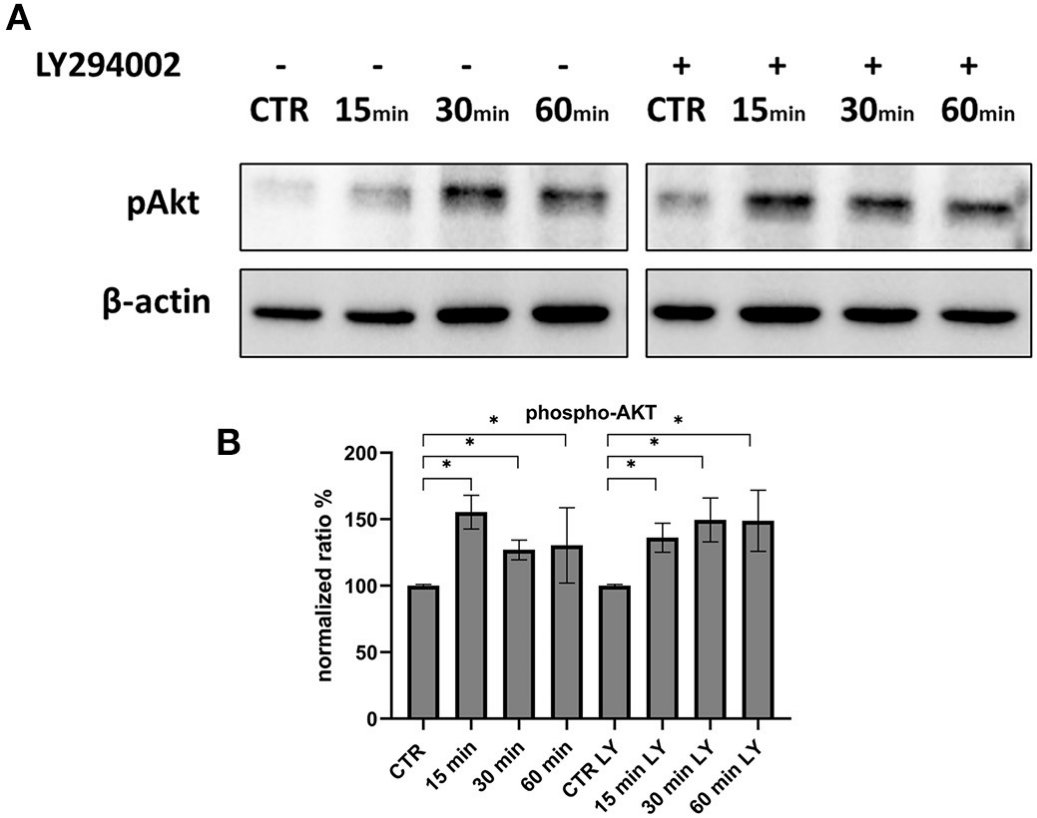
Phosphorylation of Akt during the first 60 min of infection. **(A)** Western blot: Differences in p-AKT, between control and infected cells in the presence or absence of LY294002. **(B)** Intensity of protein bands: phospho-Akt. Results were expressed as means ±SD from three independent experiments (*P < 0.05).

The effects of RAP on autophagy and expression of viral glycoproteins were also tested by Western blot (Figure 7). The results were in contrast to those obtained when early-stage inhibitors were used. Treated cells showed decreased mTOR levels at 48 and 72 h p.i. and a decrease in phospho-mTOR levels at all time points. Evidence that rapamycin-induced autophagy supported infection include increase in gB and gI expression at 24 and 48 h post infection (Figure 8).

### 3.3. Viral entry during Akt inhibition by LY294002 and Akt-1 silencing

Since Akt phosphorylation begins 3 h after infection, we investigated the very early stages of infection to determine whether there was a variation in this protein. At 15 min, p-Akt levels were higher than control, indicating that this axis was used for viral entry. The use of LY294002 during these stages did not affect the levels of phosphorylated Akt (Figure 8).

**Figure 9:**
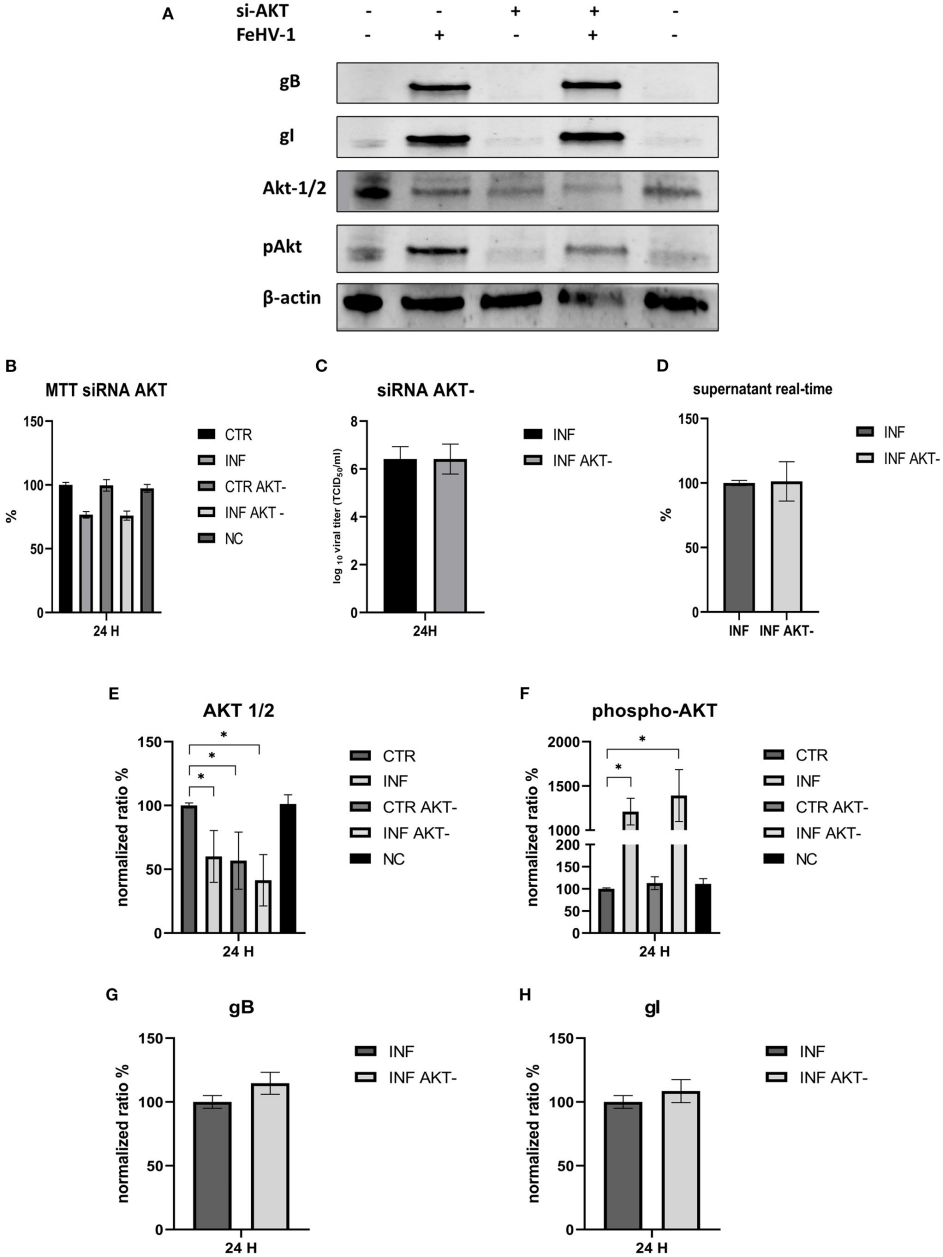
Effects of the silencing of Akt on the FeHV-1 infection. **(A)** Western blot: differences in the expression of Akt, phospho-Akt, gB, and gI among control and infected cells in presence or absence of Akt silencer. **(B)** MTT assay: Effects of Akt silencing on viability. **(C)** Effects of Akt silencing on viral titers (TCID50). **(D)** Effects of Akt silencing on viral titers (real time-PCR). **(E)** Intensity of protein bands: Akt. **(F)** Intensity of protein bands: phospho-Akt. **(G)** Intensity of protein bands: gB. **(H)** Intensity of protein bands: gI. A single representative actin has been inserted into the figure. Individual actins from each membrane, as well as full-size membranes, are available in the Supplementary material. Results were expressed as means ±SD from three independent experiments (*P < 0.05).

In another experiment, we investigated the effects of silencing Akt on infected CRFK to determine whether there were differences in the expression of autophagy markers or viral titers. Silencing was successful since Akt levels were significantly lower in silenced cells than in control cells. The p-Akt levels were lower than in infected-only cells, but viral glycoprotein expression and viral titers were comparable to those obtained in infected-only cells in both TCID_50_ and real time (Figure 9). The viability of silenced cells was not different from that of control cells, and the reduction in cell viability was consistent whether AKT-1 siRNA was present or not.

## 4. Discussion

In this work we evaluated the modification of the PI3K/Akt/mTOR axis during FeHV-1 as well as the effects on viral proliferation by inducing, the inhibiting, and silencing specific markers of this pathway. The use of multiple MOI was not associated with significant changes in Beclin-1, PI3K, Akt, or mTOR expression. We observed an increase in Akt phosphorylation in infected cells, but this was stable throughout all MOI. Phospho-mTOR was the only marker whose expression increased over time. We found no difference in UV-treated viral cells, indicating that active infection was required to modify the levels of these proteins in contrast to other viruses such as HCMV that triggers PI3K activation and Akt phosphorylation *in vitro* within the first 30 min after infection with UV-inactivated virus (12).

Our findings revealed a decrease in PI3K from 24 h after infection, and a decrease in mTOR and phospho-mTOR from 48 h p.i.. These results could be explained by the fact that the virus can interact with several markers independently, as has been demonstrated, for example, for HSV-1 and HCMV in previous works. HSV-1 was found to induce autophagy in glioma cells without a significant increase in the expression of the pro-autophagic protein Beclin-1 and an increase in the phosphorylation of mTOR and Akt (24). Moreover, these results, would indicate the inhibition of autophagy pathways other than the ones it triggers.

We can also assume that the phosphorylation of Akt is triggered during the viral entry; in fact, in the first moment, we found significantly higher levels of phospho-Akt 3 h after infection (when no other markers showed any modifications) as well as after 15 min from the adsorption. Phosphorylation of Akt was stable for all the times of infection studied. Furthermore, constitutive Akt was reduced only 12 h after infection. A possible explanation for these results may be the involvement of Akt phosphorylation in viral entry, as has already been demonstrated for several herpesviruses such as HSV-1, DEV, and PRV (8, 11, 23, 25, 26). For example, during HSV-1 infection, activation of the PI3K/Akt phosphorylation cascade is most likely due to the actions of the VP11/12 protein, through binding of the virus to the cell surface, while maintenance is preserved by US3 (27). This viral kinase is able to phosphorylate multiple Akt substrates as a replacement for Akt. The importance of the US3 protein has been also demonstrated for DEV and PRV, which share several characteristics with FeHV-1. The presence of a region encoding US3 has already been identified in the FeHV-1 genome, as well as a recent work has demonstrated its importance for viral replication (deletion of the UL3 gene significantly reduces FeHV-1 virulence) (28). Furthermore, US3 of FeHV-1 shows similarity to US3 expressed by DEV (about 67% percent sequence identity) and HSV-1 (approximately 65% sequence identity). Based on these analogies and the results obtained by silencing Akt-1 and using Akt inhibitors (such as LY294002), we can hypothesize that the virus uses the virally encoded Akt primarily for the first 6 h after infection, which is sufficient to ensure an effective infection. The virus would then use both its own and cellular Akt. In fact, the silencing of Akt did not affect the viral titers, as well as the use of LY294002 in the first stages of infection did not prevent the Akt phosphorylation. Also, HSV-1 induces Akt phosphorylation within minutes, but in this case, chemical inhibition of PI3K activity with LY294002 or the use of Akt silencers blocked HSV entry and fusion. The ability of Akt-1 silencing and LY294002 treatment to inhibit viral proliferation may differ due to the diversity of Akt isoforms. Even though Akt-1 silencing has been shown to be the most effective in reducing viral titer in some viruses, such as HSV, we cannot exclude the possibility that other Akt isoforms, where they have not been silenced (there is a high degree of homology among the various isoforms), may have contributed to supply Akt (23). Early stage autophagic inhibitors had no discernible effect on viral titres, but in a previous work late-stage inhibitors drastically reduced viral yields. In fact, only transient reduced viral titres and glycoproteins expression was observed (12 h for LY294002 and 48 h for 3-MA). The results obtained by us regarding the effects of early-stage autophagy inhibitors on viral replication differ from those obtained in previous research that assessed the effects of the same chemicals on viral proliferation. For example, during DEV and VZV infection, LY treatment lowered viral titer, whereas 3-MA treatment reduced viral titer during PRV infection (9, 20, 29). On the other hand, our results show that the use of RAP enhances viral proliferation, and this mechanism has been described previously for other herpesviruses DEV and PRV (20, 29).

Several viruses other than Herpesvirus can interact with PI3K/AKT/mTOR axis. Examples are Dengue virus (DENV) that impairs this pathway, porcine circovirus type 2 (PCV-2), which can transiently induce the PI3K/Akt/mTOR axis (also by UV-treated virus), or PEDV that, thanks to nsp6, can induce autophagy mainly via this signaling (16, 17, 30). Other examples include rotavirus, porcine parvovirus, duck tembusu virus, and Marek disease virus, all of which can induce autophagy *via* the PI3K–AKT–mTOR pathway (15, 31–33). On the other hand, human papillomavirus activates the PI3K/Akt/mTOR signaling to inhibit autophagy in the early stages of virus-host cell interactions (34). Therefore, we can assume that the same event (activation of the PI3K–AKT–mTOR pathway) could result in different implications.

Many viruses have specific proteins that can stimulate the axis, such as VP12 for VZV or VP11/12 and US3 protein kinase for HSV-1 (10). Further research on the deletion of Us3 during FeHV-1 should be conducted to assess the effects on autophagy and the PI3K/AKT/mTOR axis, given that a recent study demonstrated the importance of this protein for viral replication (the deletion blocked viral proliferation).

A further research topic would be the establishment of the relationship among other important cellular pathways (such as apoptosis or the expression of proteins involved in the interferon response), which we tend to formally distinguish but which biologically overlap and are interconnected.

## Data availability statement

The original contributions presented in the study are included in the article/Supplementary material, further inquiries can be directed to the corresponding author.

## Author contributions

GF: experimental design, data collection, and analyses. GF, UP, and SM: manuscript writing. UP, SD, SM, RC, and CL: conception of the study, manuscript editing, data visualization, and statistical analysis. All authors contributed to the article and approved the submitted version.
