# Supplementary material for: Modifications of the PI3K/Akt/mTOR axis during FeHV-1 infection in permissive cells
Source: Front Vet Sci. 2023 Mar 21;10:1157350. doi: 10.3389/fvets.2023.1157350 (PMC10072329; doi:10.3389/fvets.2023.1157350)
Supplement: Supplementary file 1 [file Data_Sheet_1.PDF]

Fig 1

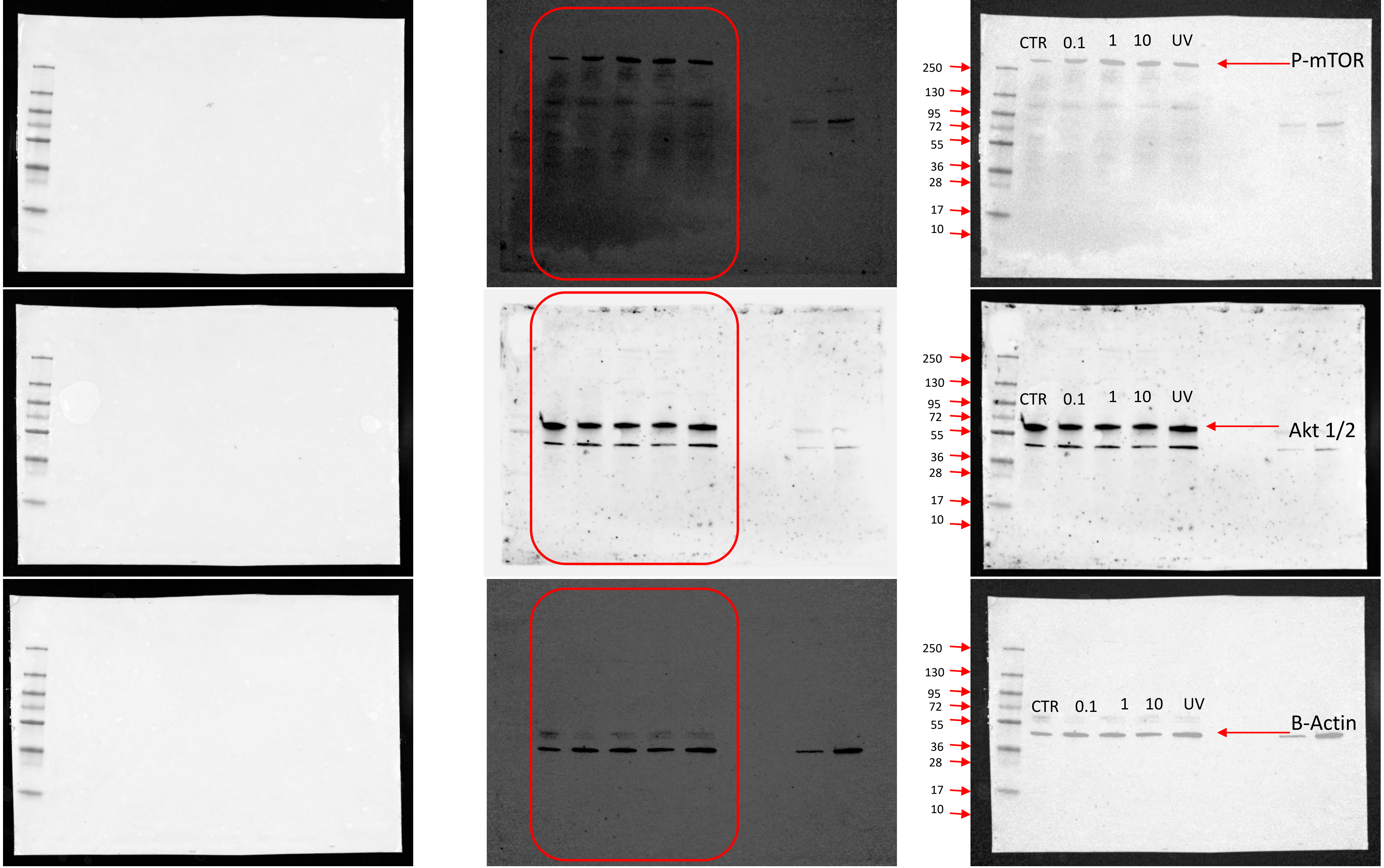

CTR 0.1 1 10 UV
P-mTOR
250
130
95
72
55
36
28
17
10
CTR 0.1 1 10 UV
Akt 1/2
250
130
95
72
55
36
28
17
10
CTR 0.1 1 10 UV
B-Actin
250
130
95
72
55
36
28
17
10

Fig 1

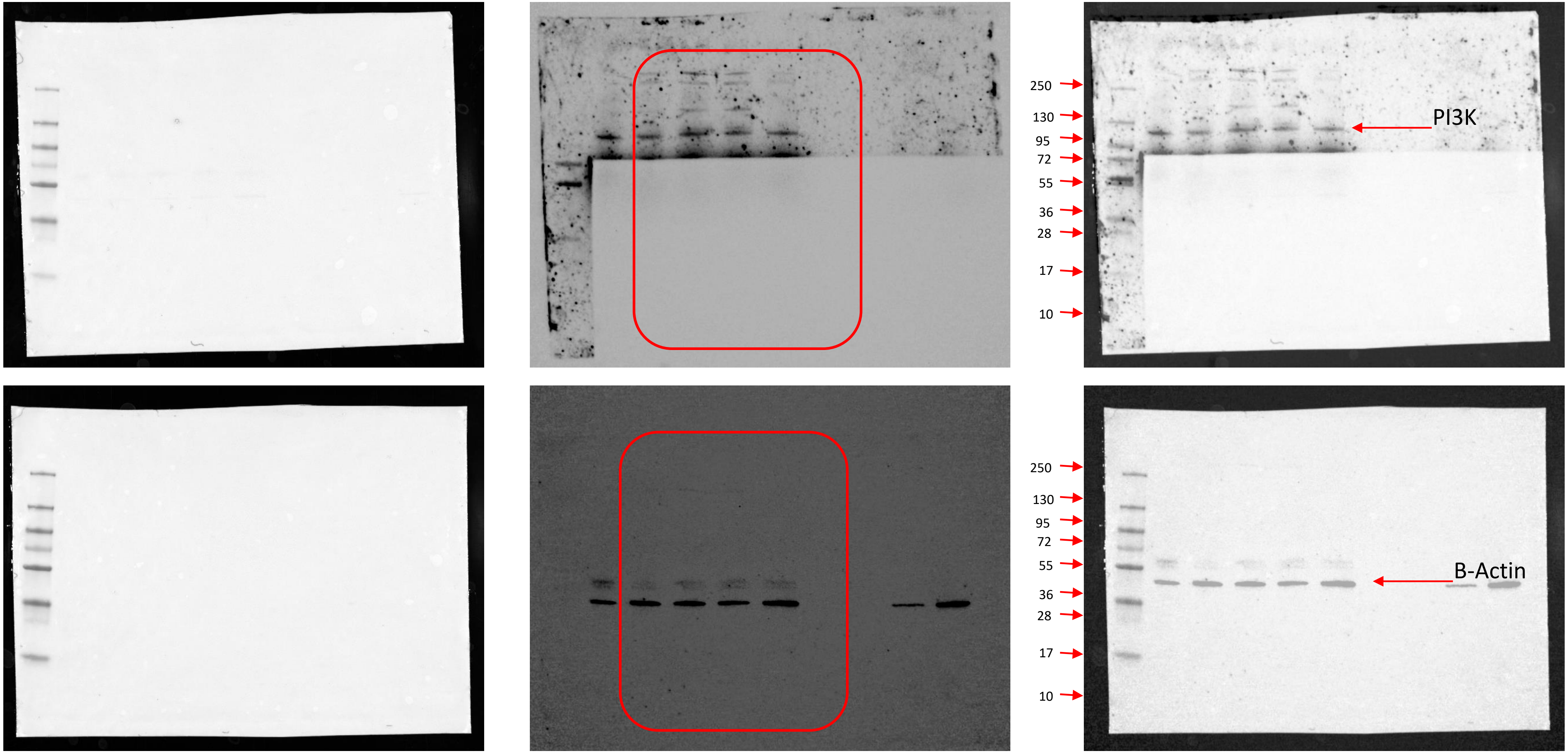
250
130
95
72
55
36
28
17
10
PI3K
250
130
95
72
55
36
28
17
10
B-Actin

Fig 1

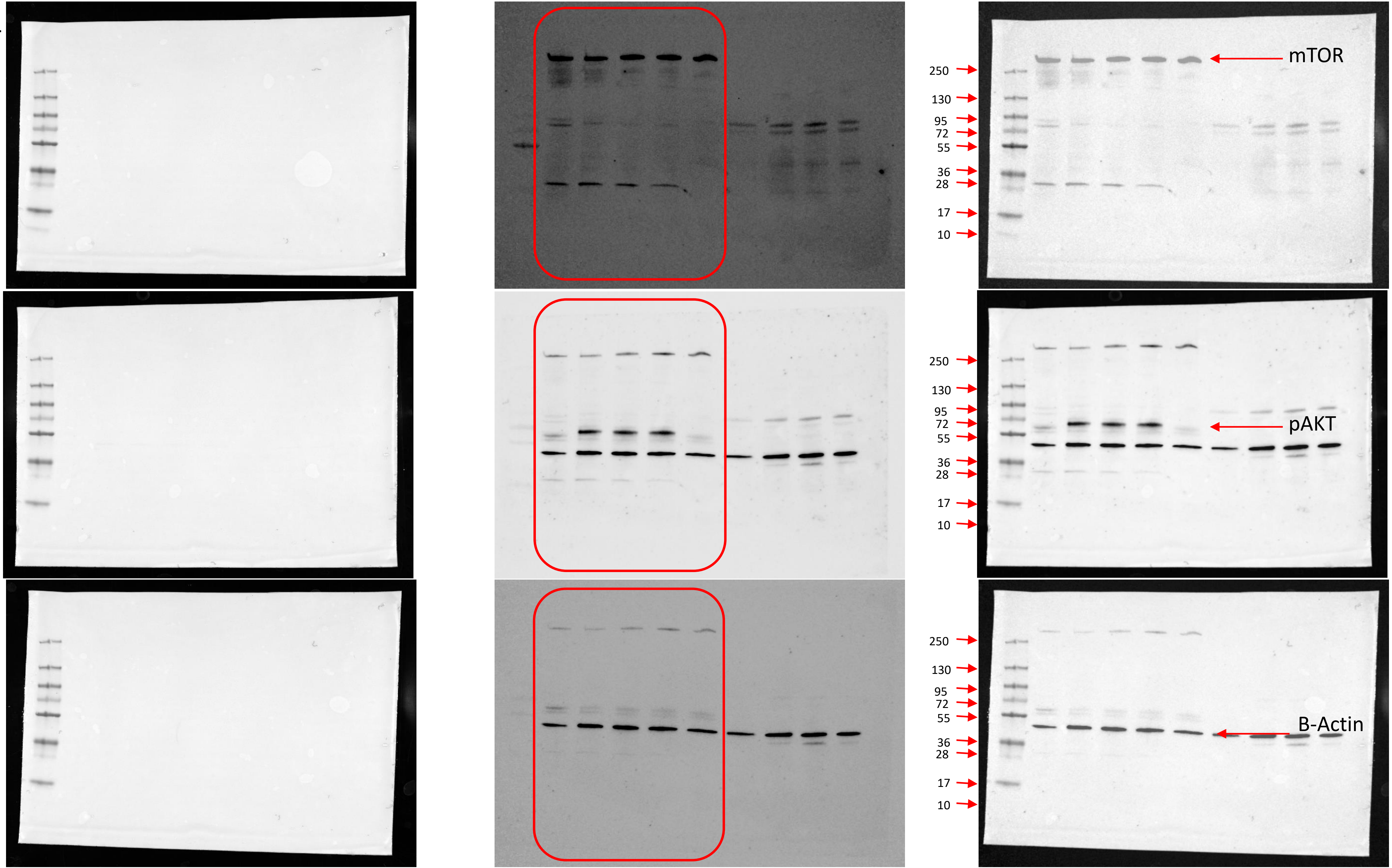

Fig 1

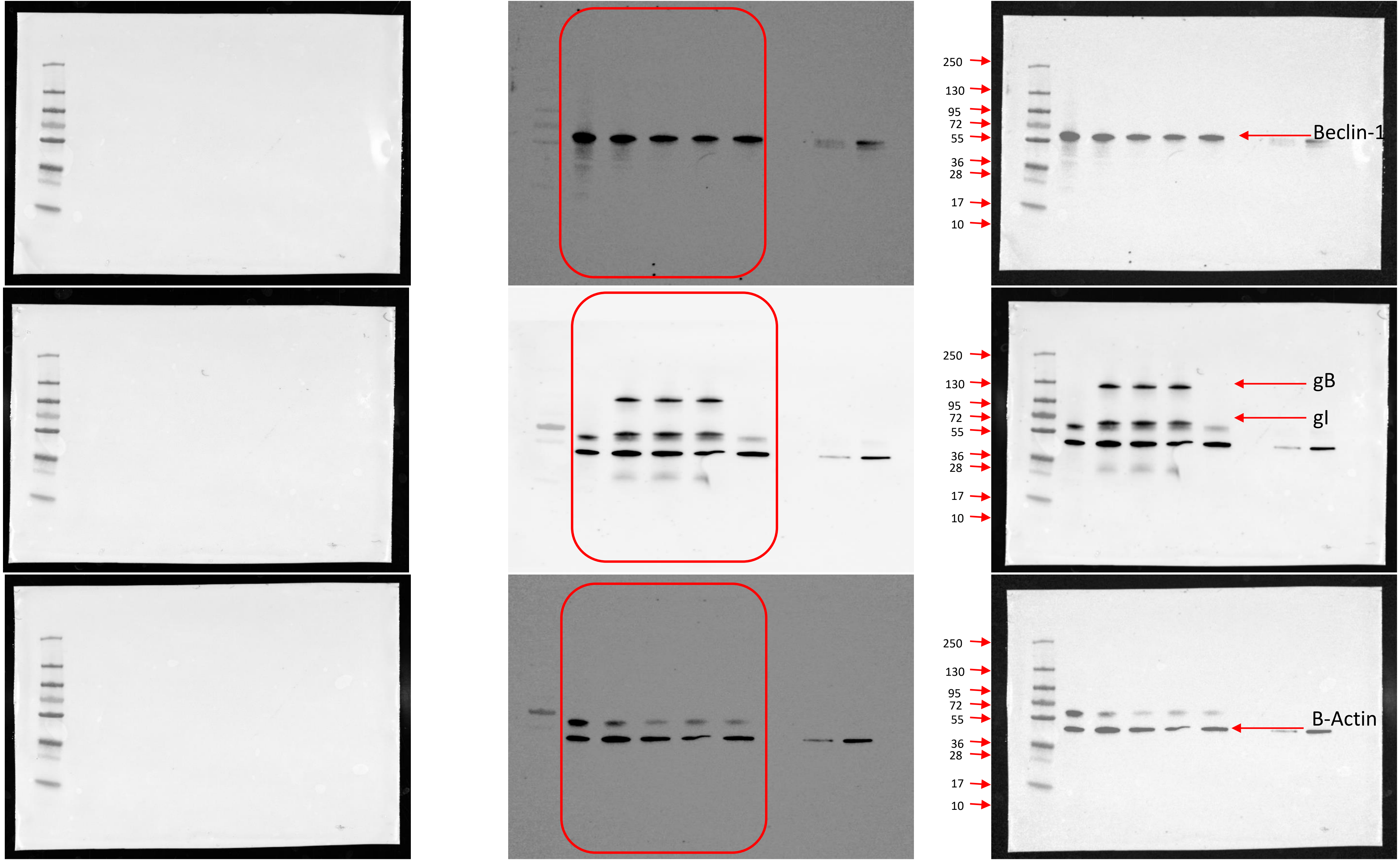

Fig 2

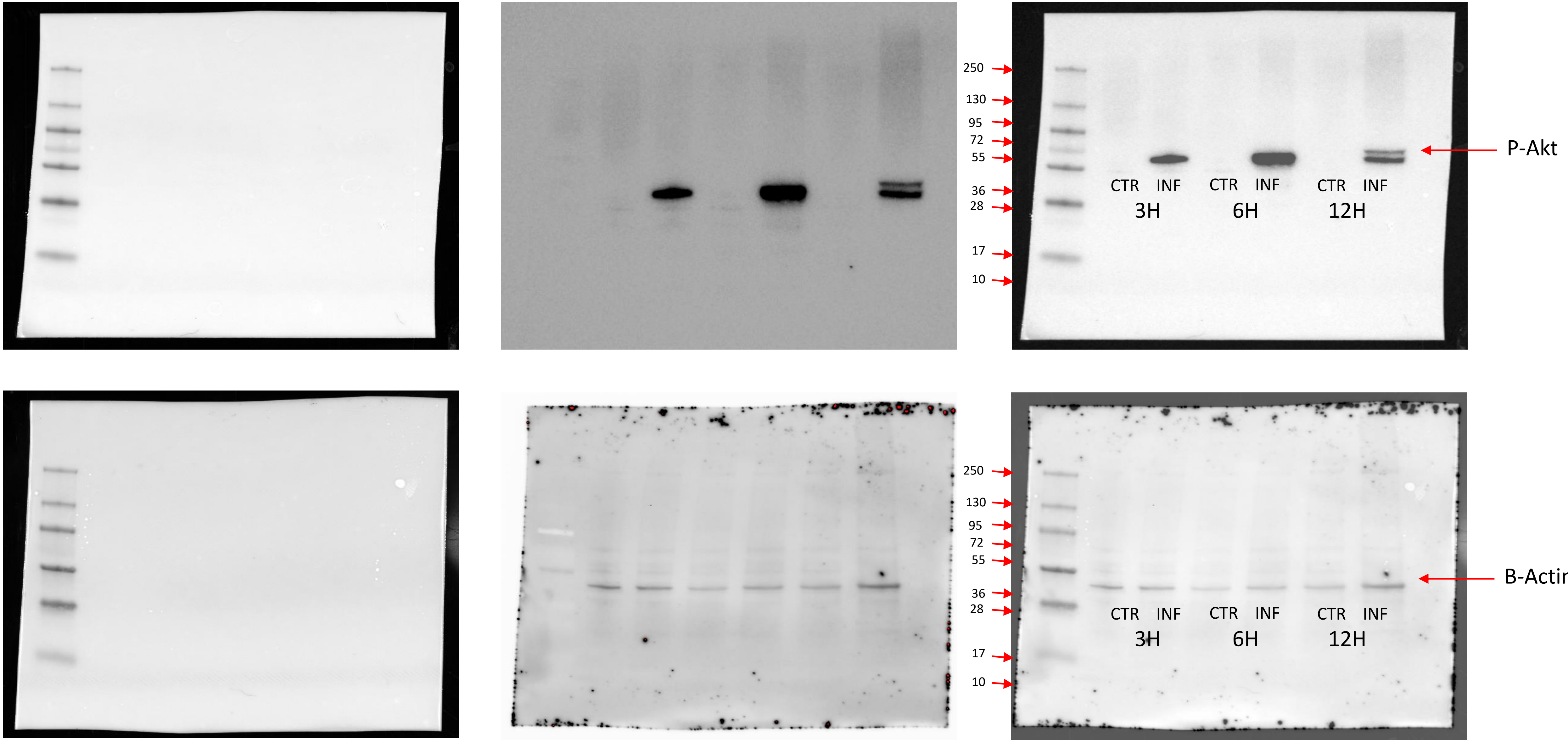

250
130
95
72
55
36
28
17
10
P-Akt
CTR INF CTR INF CTR INF
3H 6H 12H
250
130
95
72
55
36
28
17
10
B-Acti
CTR INF CTR INF CTR INF
3H 6H 12H

Fig 2

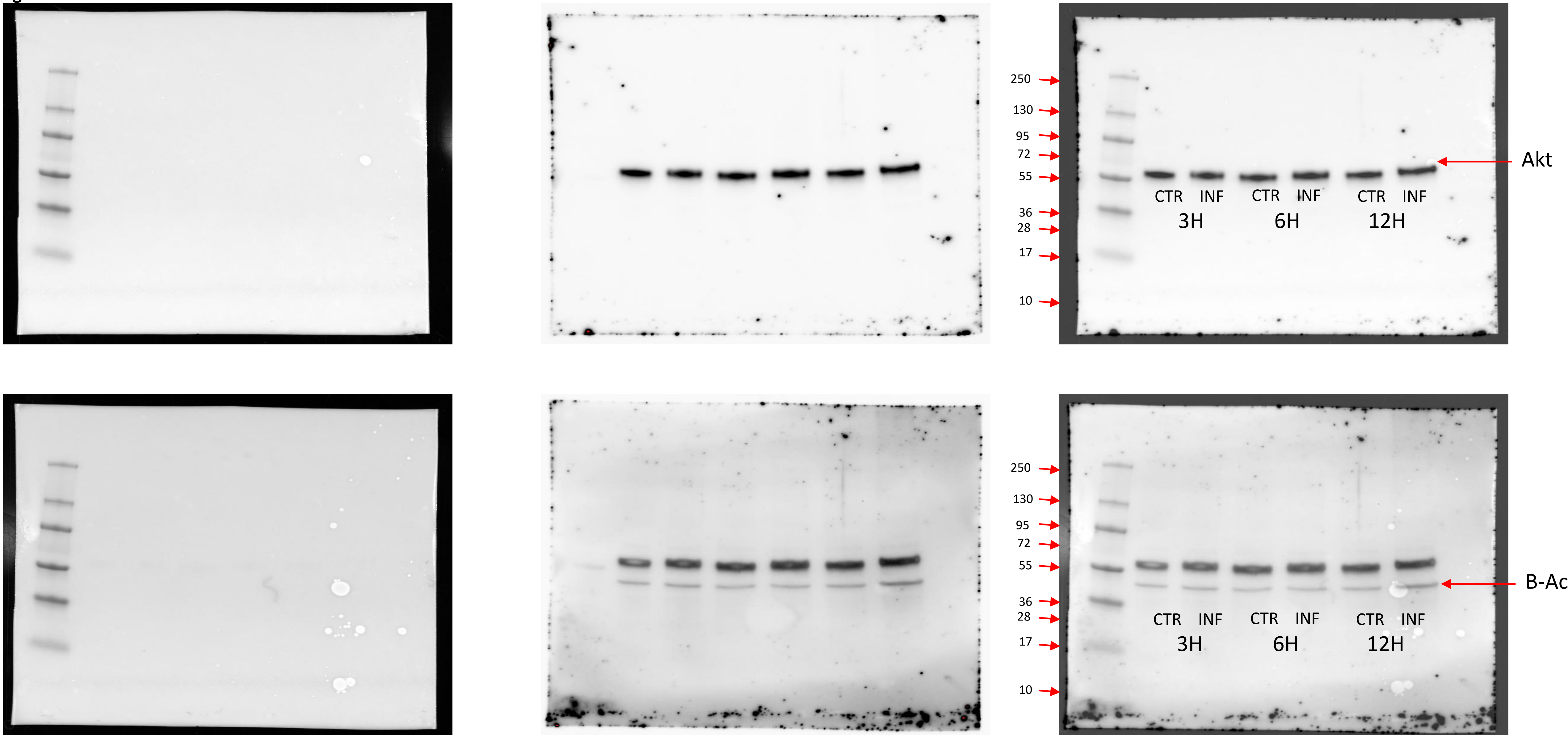

250
130
95
72
55
36
28
17
10
Akt
CTR
INF
CTR
INF
CTR
INF
3H
6H
12H
250
130
95
72
55
36
28
17
10
B-Ac
CTR
INF
CTR
INF
CTR
INF
3H
6H
12H

Fig 2

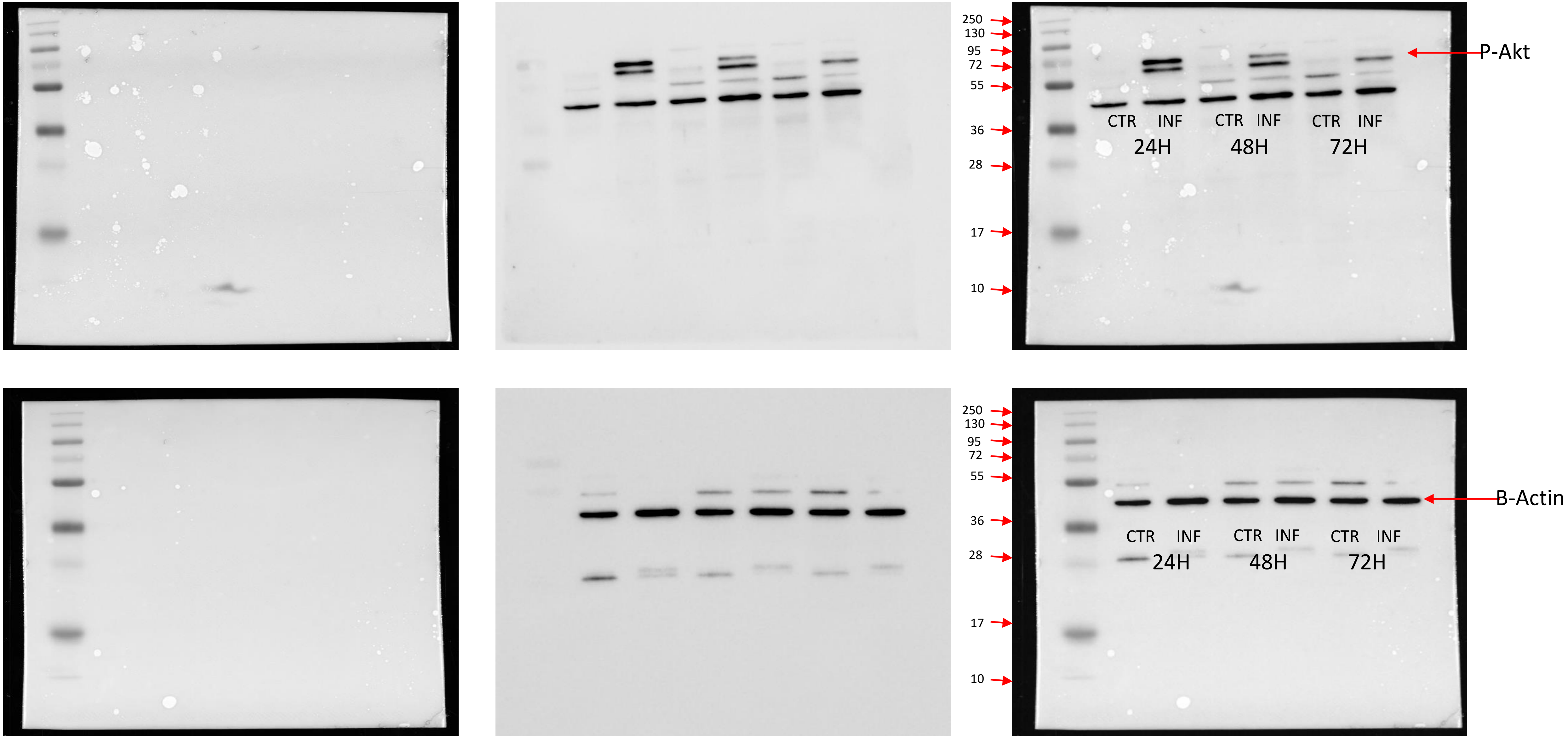

250
130
95
72
55
36
28
17
10
P-Akt
CTR
INF
CTR
INF
CTR
INF
24H
48H
72H
250
130
95
72
55
36
28
17
10
B-Actin
CTR
INF
CTR
INF
CTR
INF
24H
48H
72H

Fig 2

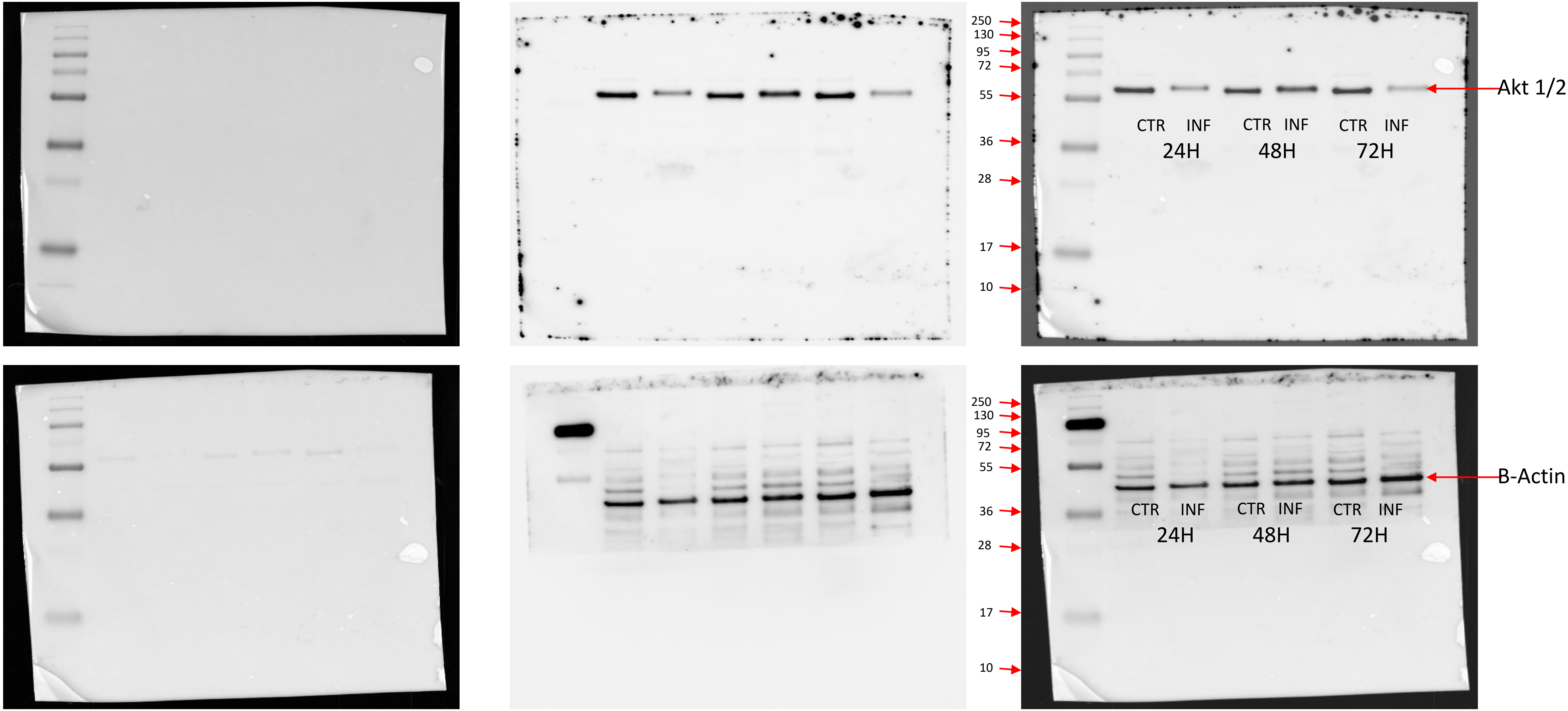

250
130
95
72
55
36
28
17
10
Akt 1/2
CTR
INF
CTR
INF
CTR
INF
24H
48H
72H
250
130
95
72
55
36
28
17
10
B-Actin
CTR
INF
CTR
INF
CTR
INF
24H
48H
72H

Fig 2

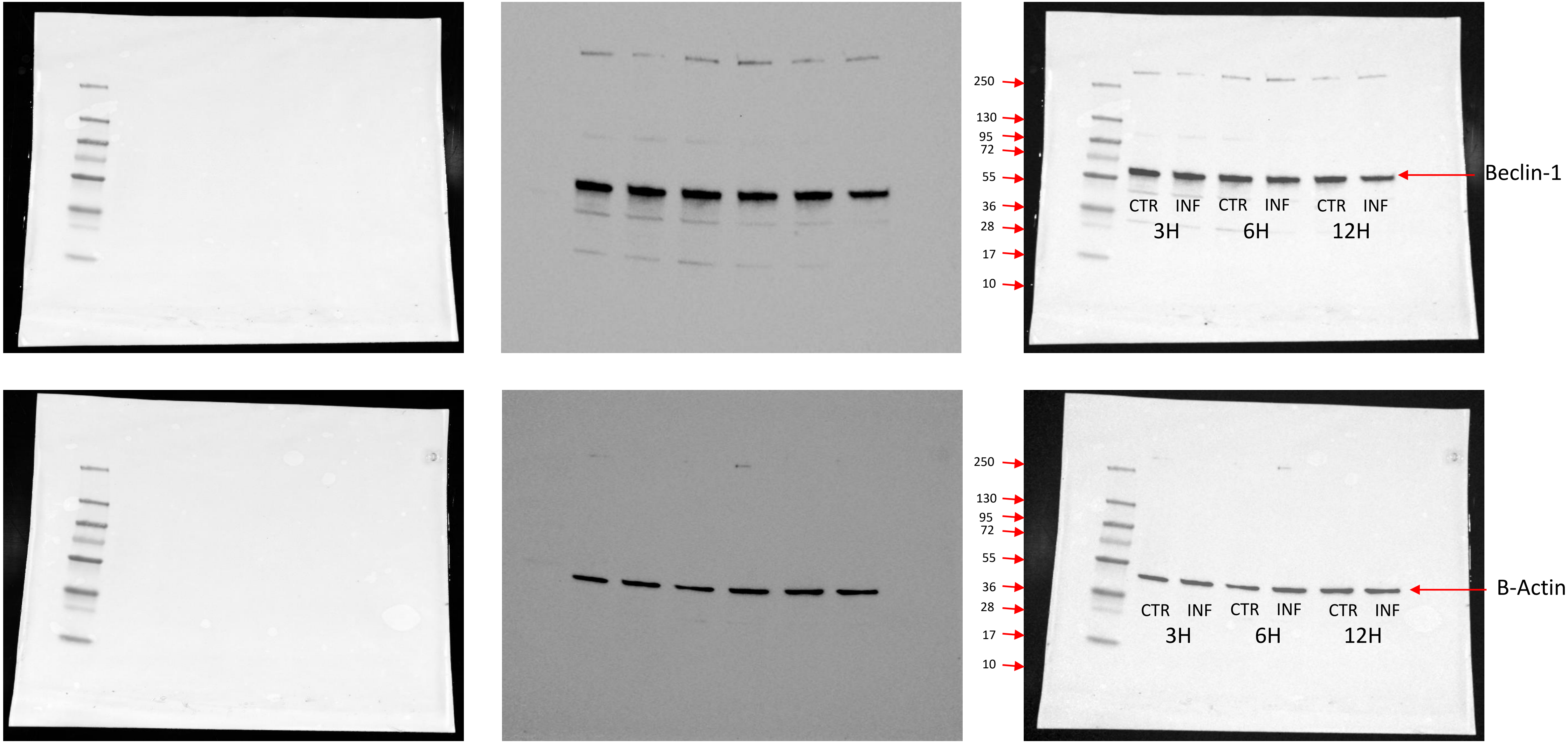
250
130
95
72
55
36
28
17
10
Beclin-1
CTR INF CTR INF CTR INF
3H 6H 12H
250
130
95
72
55
36
28
17
10
B-Actin
CTR INF CTR INF CTR INF
3H 6H 12H

Fig 3

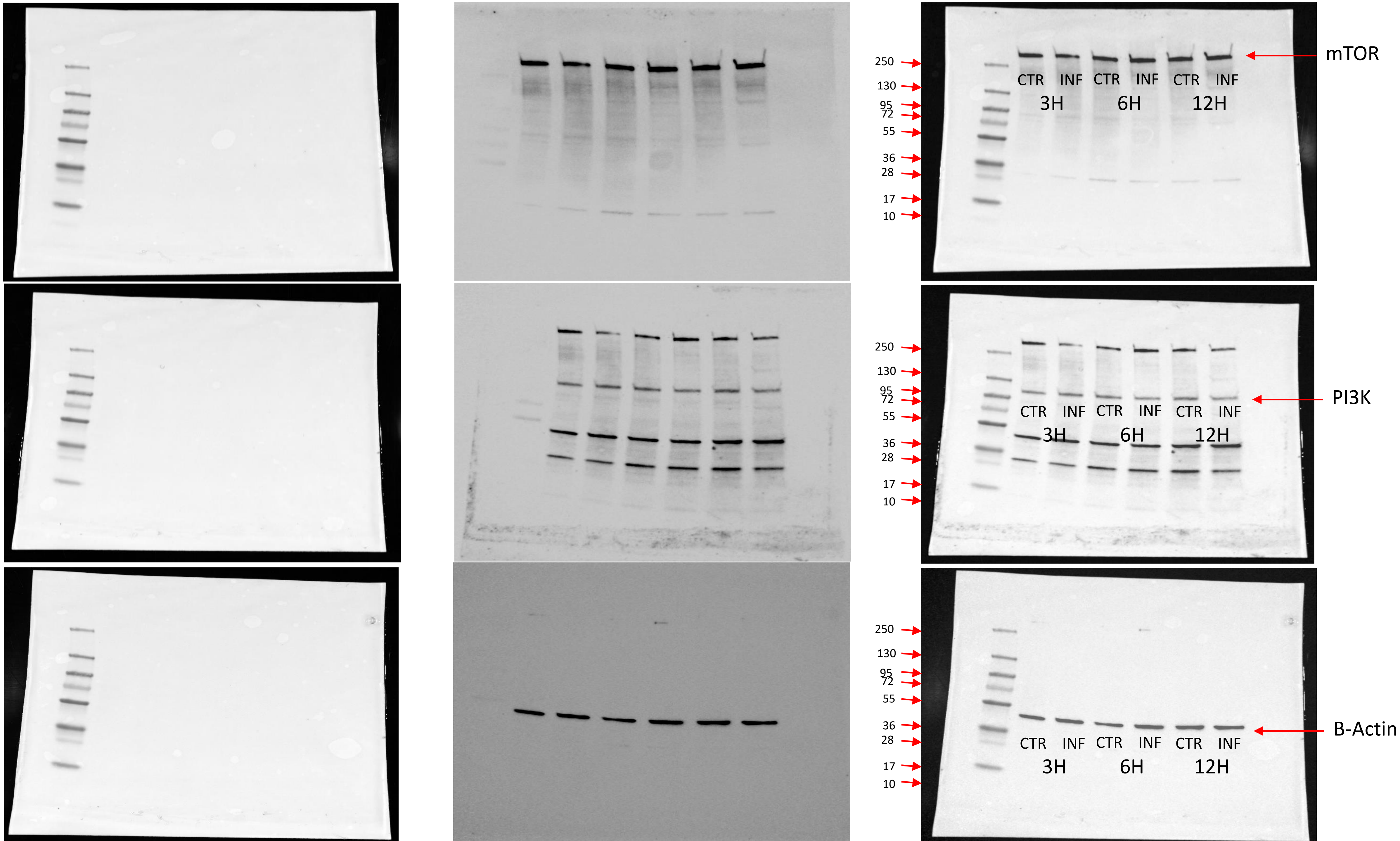

250
130
95
72
55
36
28
17
10
mTOR
CTR INF CTR INF CTR INF
3H 6H 12H
PI3K
B-Actin

Fig 3

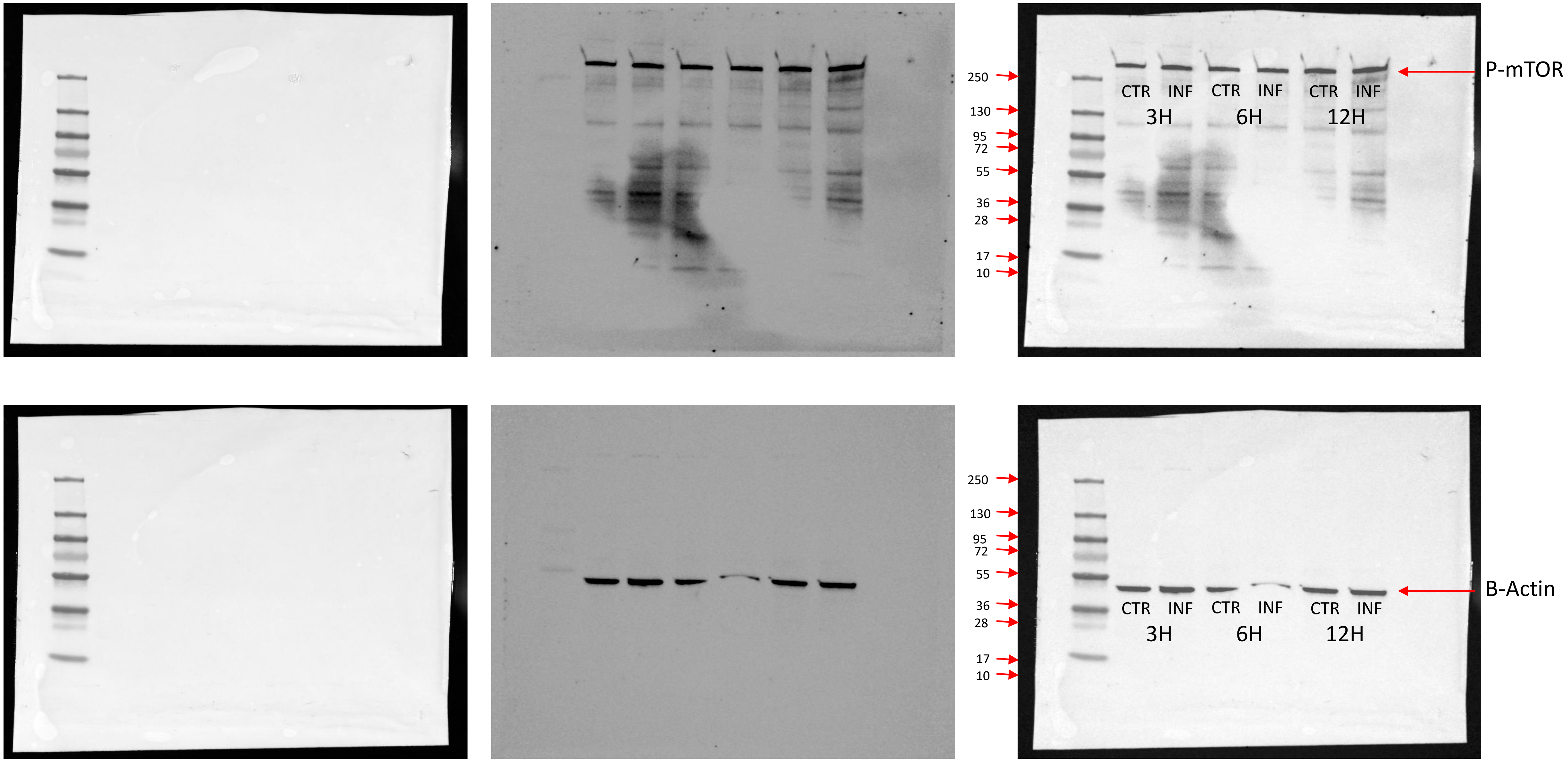
250
130
95
72
55
36
28
17
10
CTR
INF
CTR
INF
CTR
INF
3H
6H
12H
P-mTOR
250
130
95
72
55
36
28
17
10
CTR
INF
CTR
INF
CTR
INF
3H
6H
12H
B-Actin

Fig 3

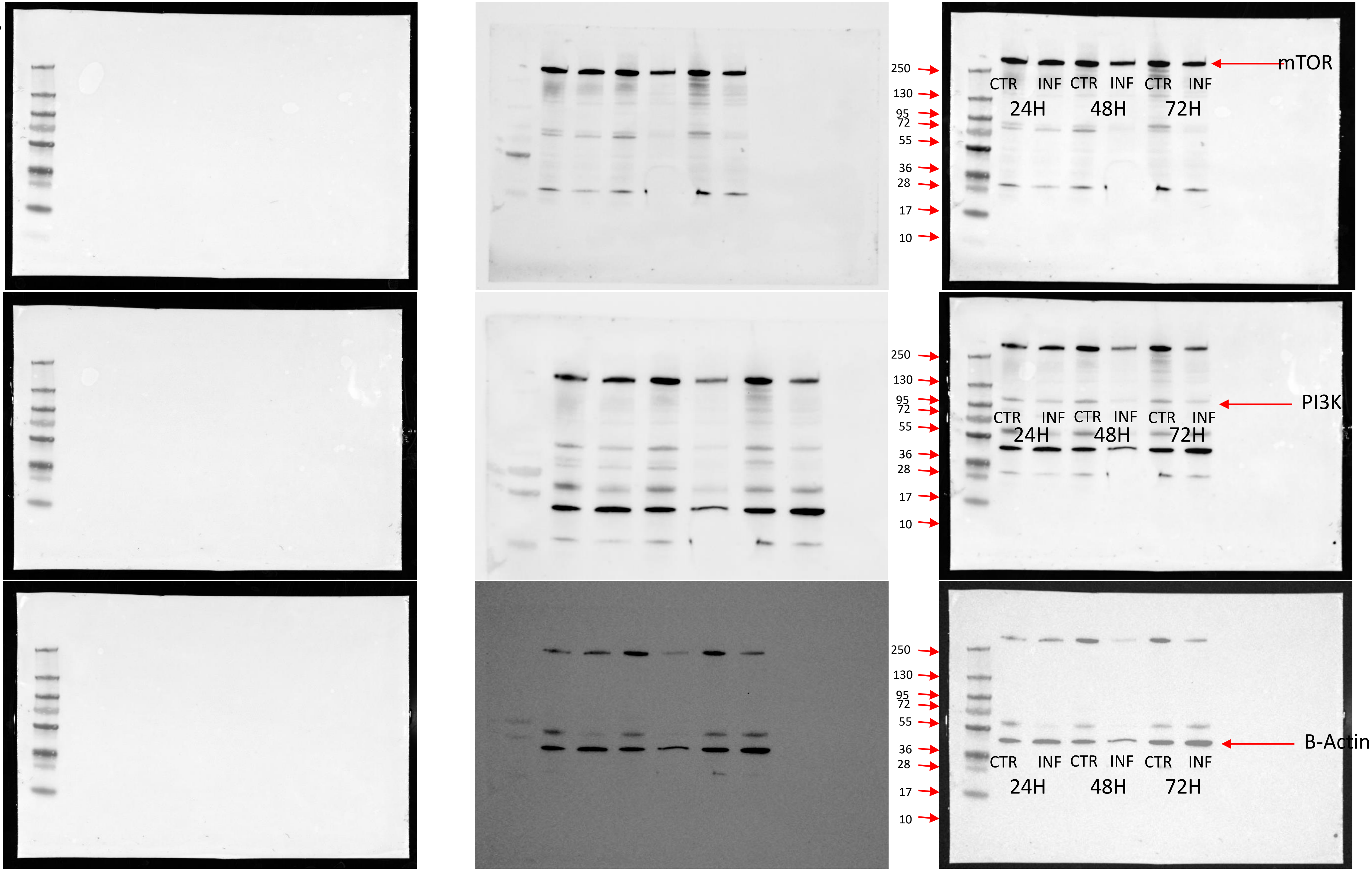
250
130
95
72
55
36
28
17
10
mTOR
CTR INF CTR INF CTR INF
24H 48H 72H
250
130
95
72
55
36
28
17
10
PI3K
CTR INF CTR INF CTR INF
24H 48H 72H
250
130
95
72
55
36
28
17
10
B-Actin
CTR INF CTR INF CTR INF
24H 48H 72H

Fig 3

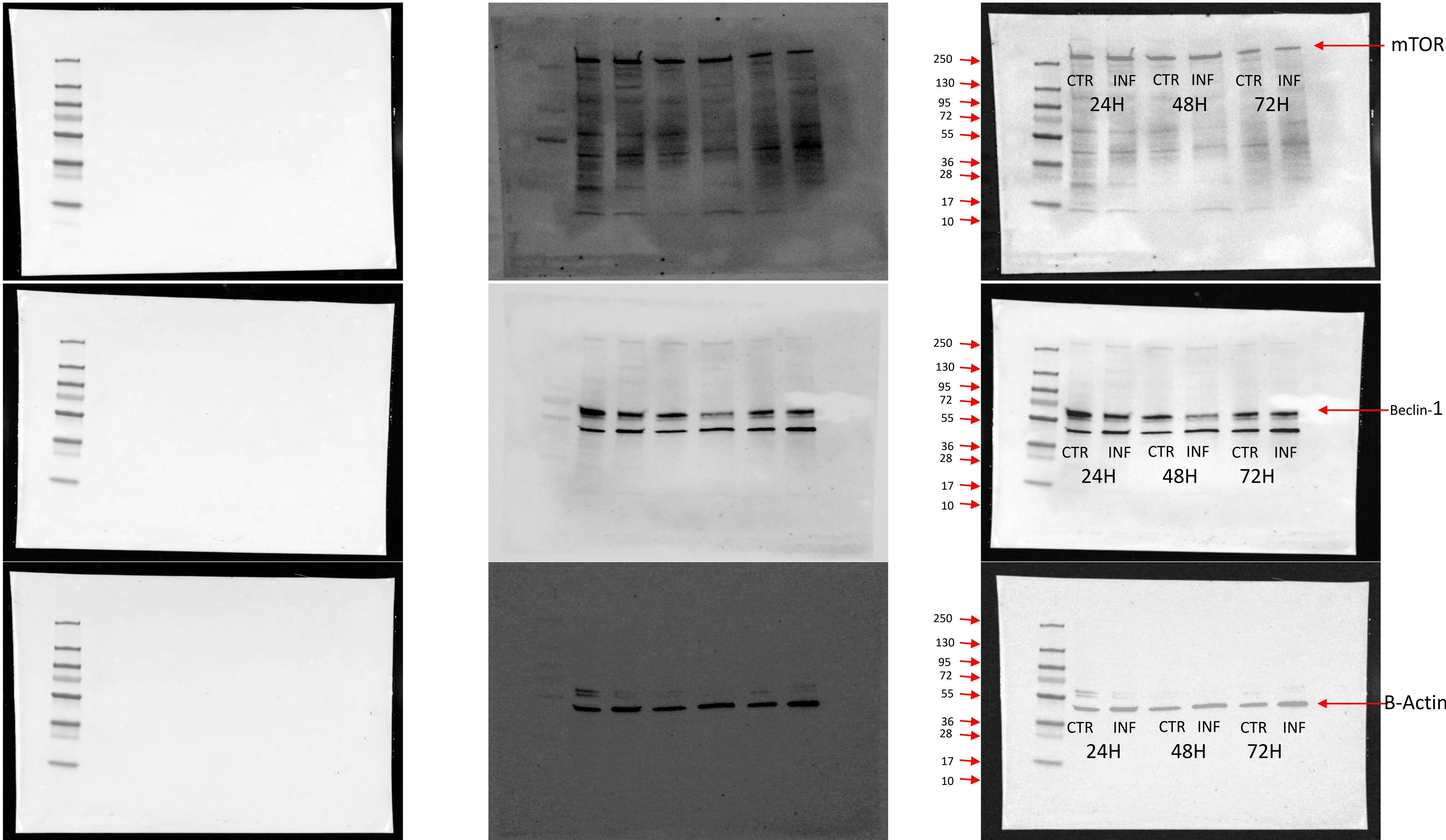
mTOR
Beclin-1
B-Actin
CTR
INF
CTR
INF
CTR
INF
24H
48H
72H
250
130
95
72
55
36
28
17
10

Fig 5

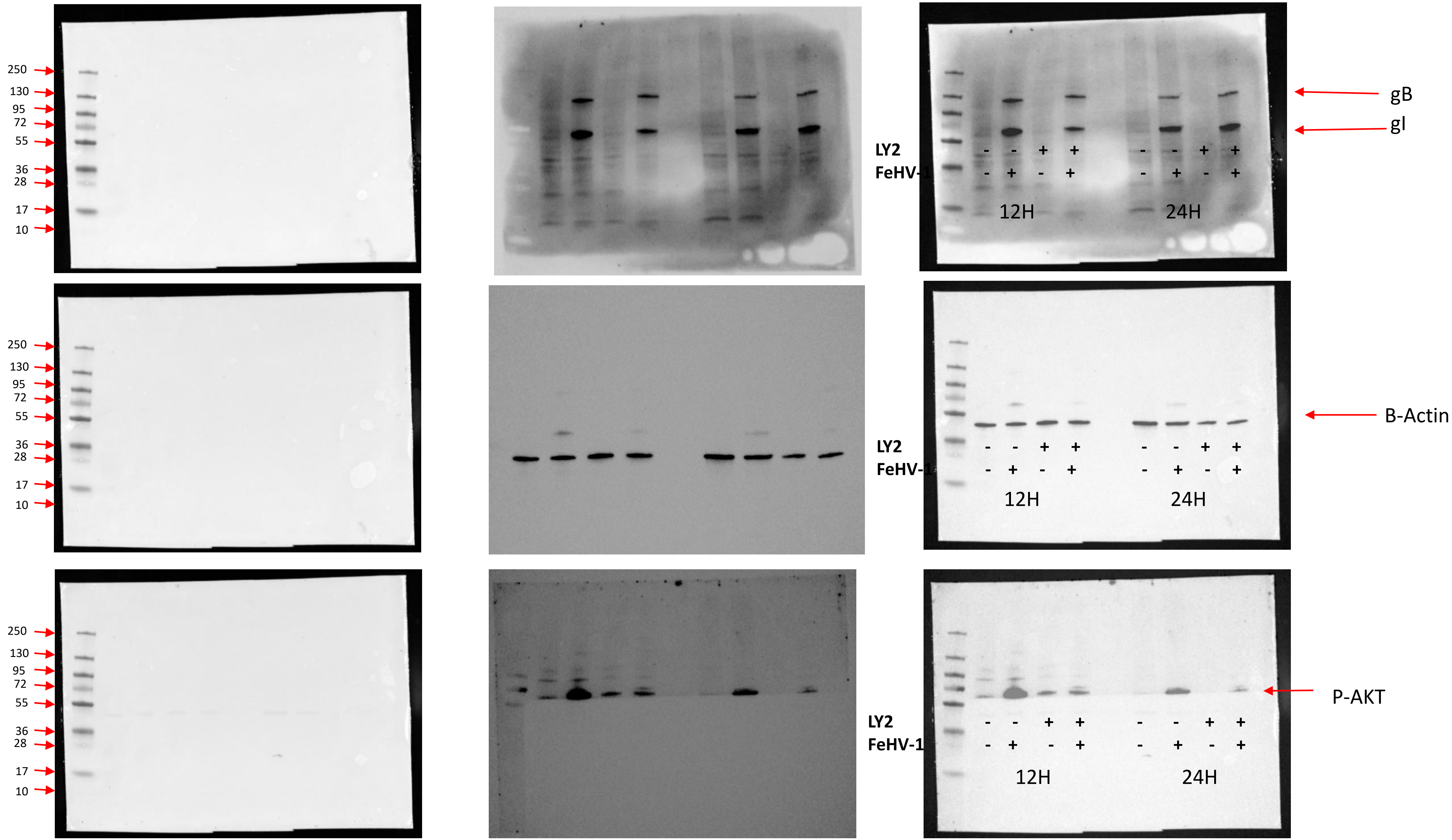
250
130
95
72
55
36
28
17
10
gB
gI
LY2 - - + + - - + +
FeHV-1 - + - + - + - +
12H
24H
B-Actin
LY2 - - + + - - + +
FeHV-1 - + - + - + - +
12H
24H
P-AKT
LY2 - - + + - - + +
FeHV-1 - + - + - + - +
12H
24H

Fig 5

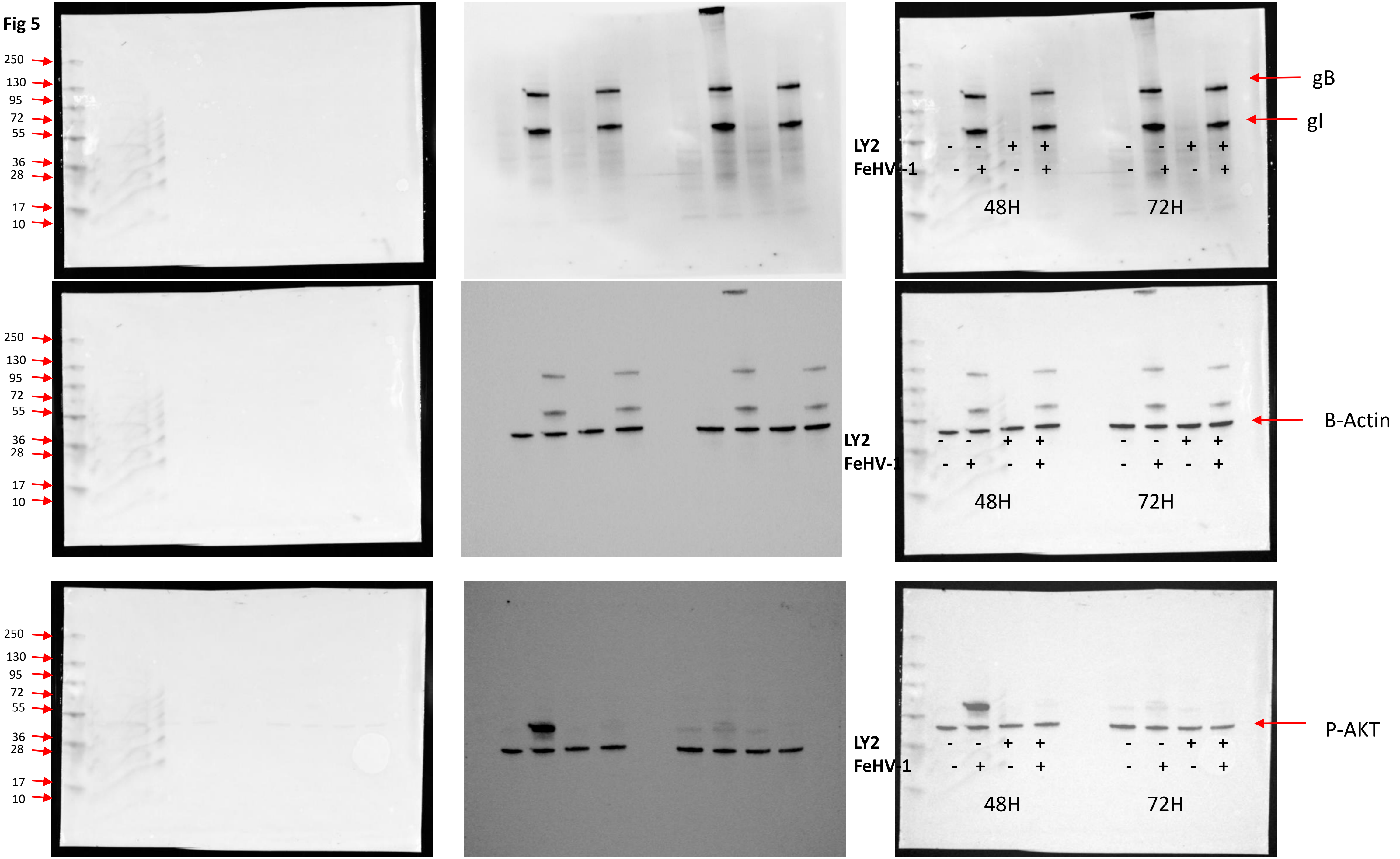

Fig 6

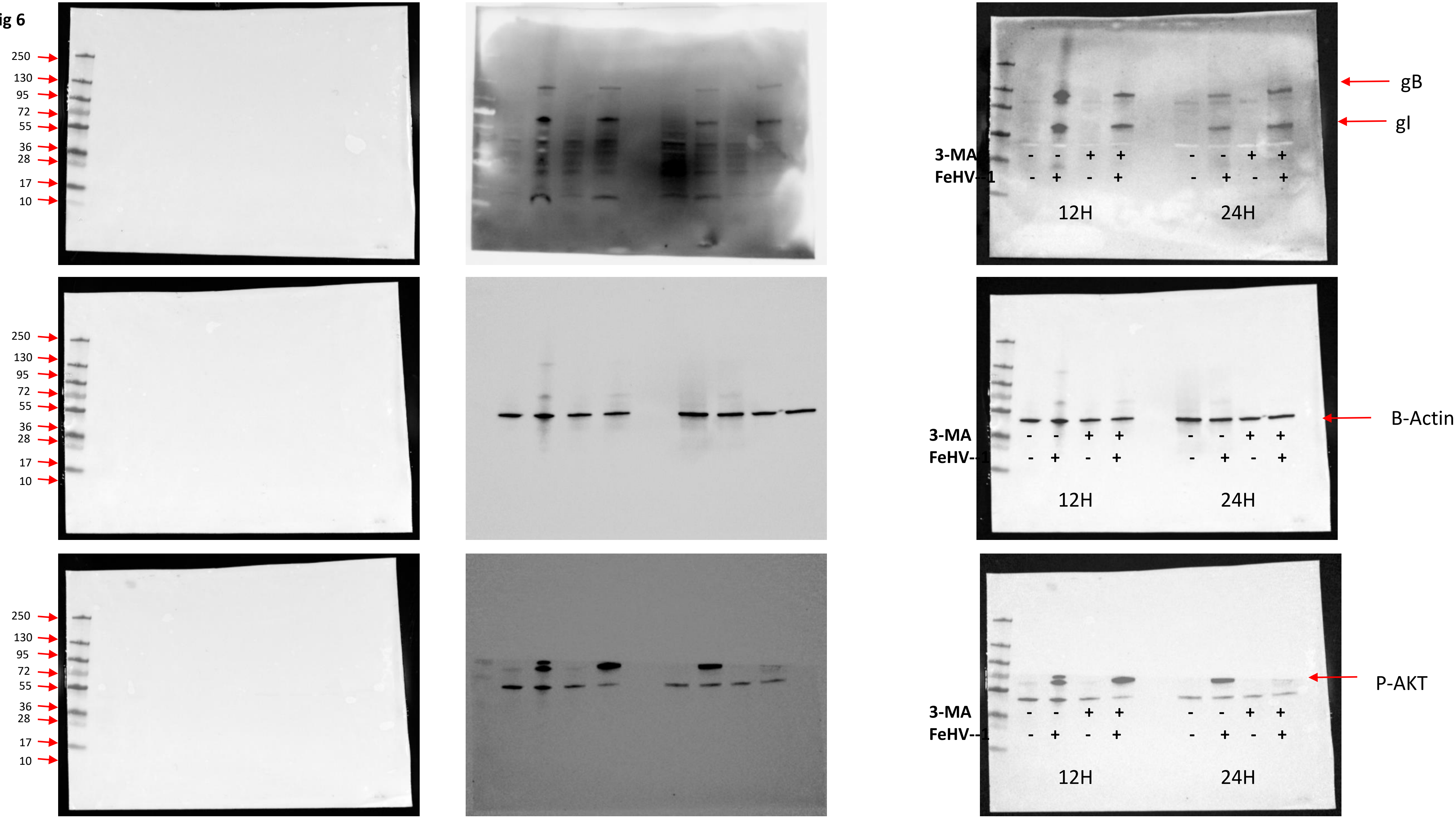

Fig 6

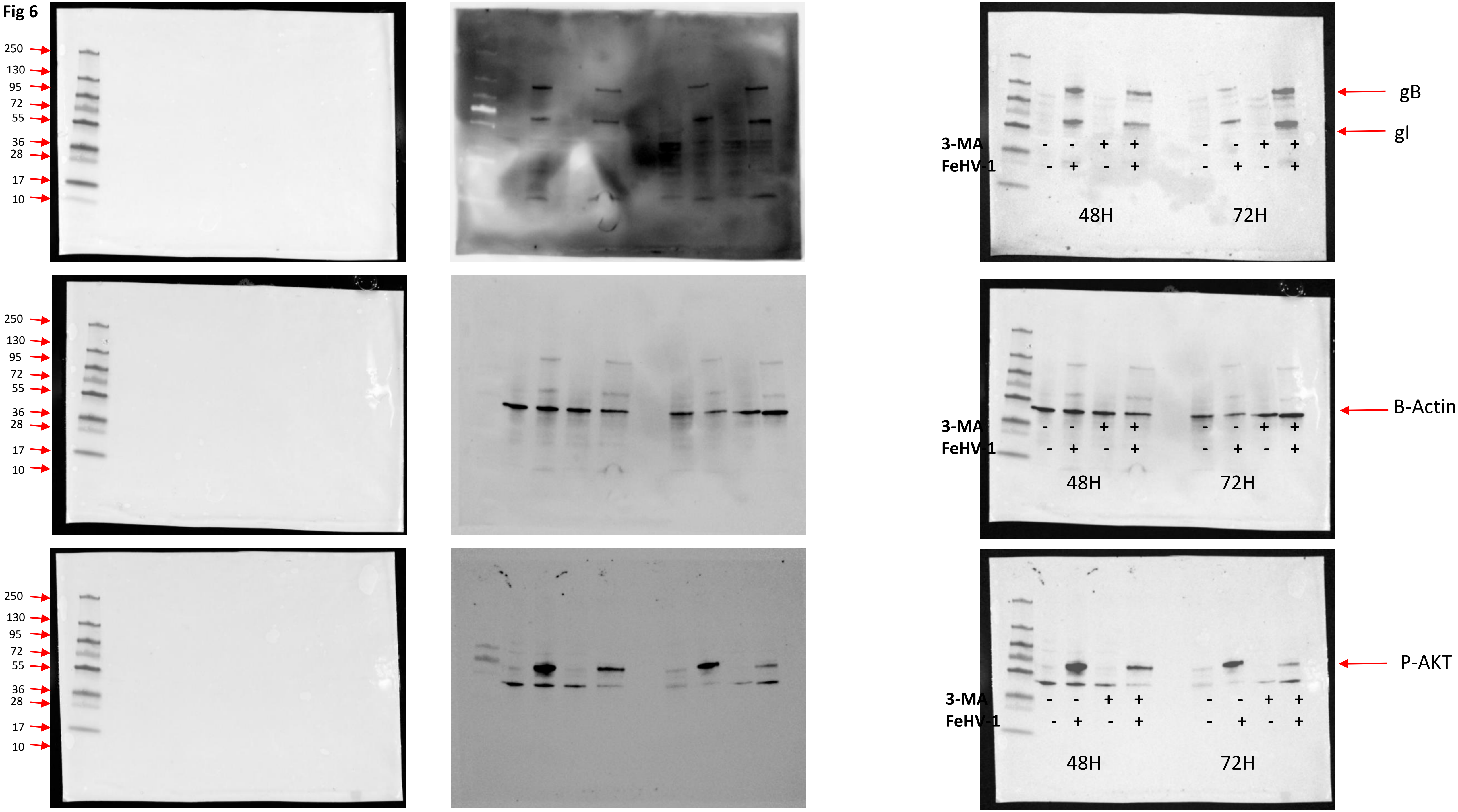

Fig 6

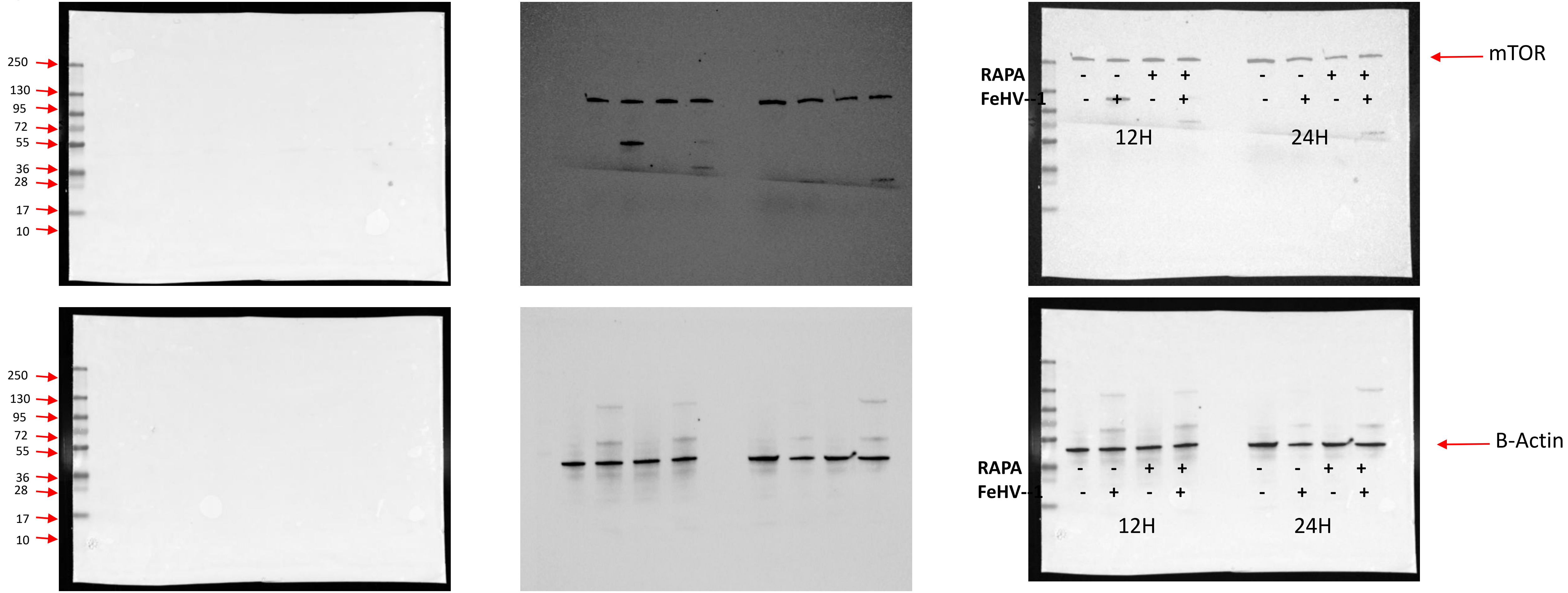

250
130
95
72
55
36
28
17
10
RAPA - - + + - - + +
FeHV--1 - + - + - + - +
12H
24H
mTOR
250
130
95
72
55
36
28
17
10
B-Actin
RAPA - - + + - - + +
FeHV--1 - + - + - + - +
12H
24H

Fig 7

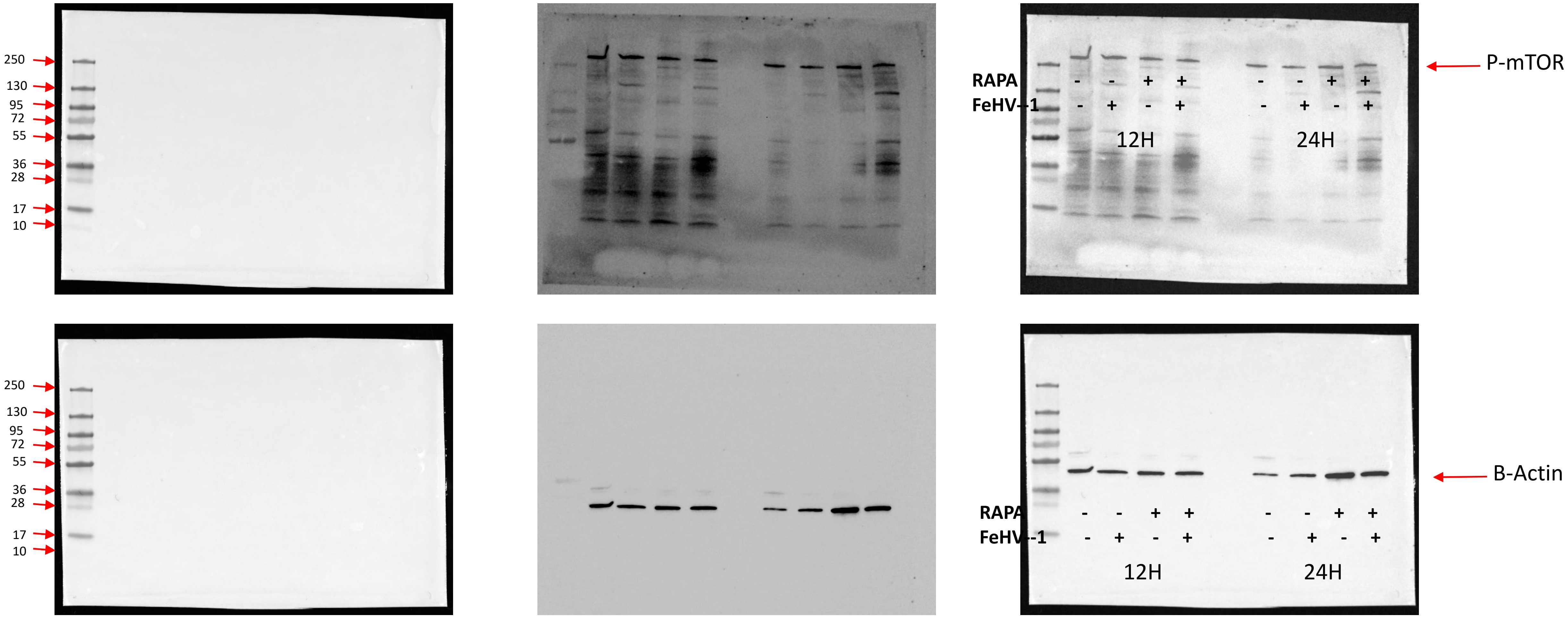
250
130
95
72
55
36
28
17
10
RAPA - - + + - - + +
FeHV--1 - + - + - + - +
12H
24H
P-mTOR
250
130
95
72
55
36
28
17
10
RAPA - - + + - - + +
FeHV--1 - + - + - + - +
12H
24H
B-Actin

Fig 7

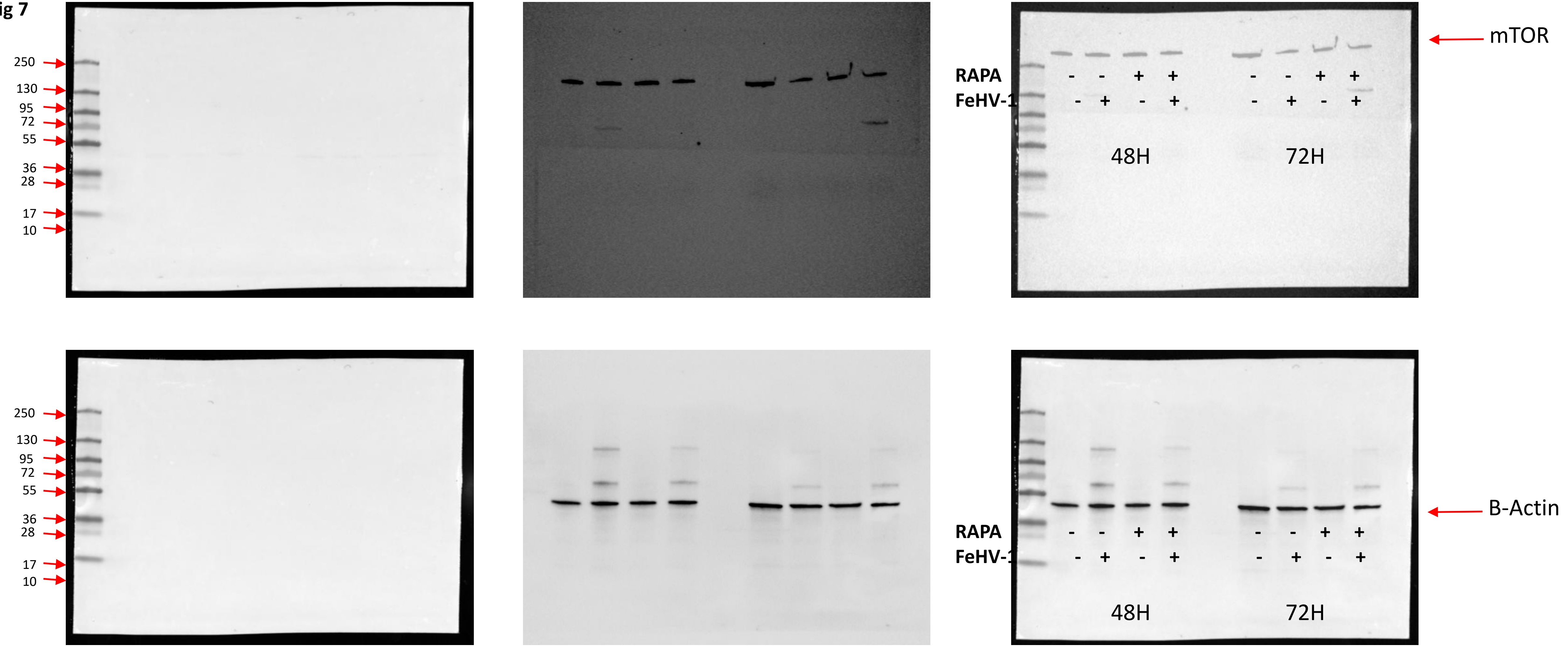

Fig 7

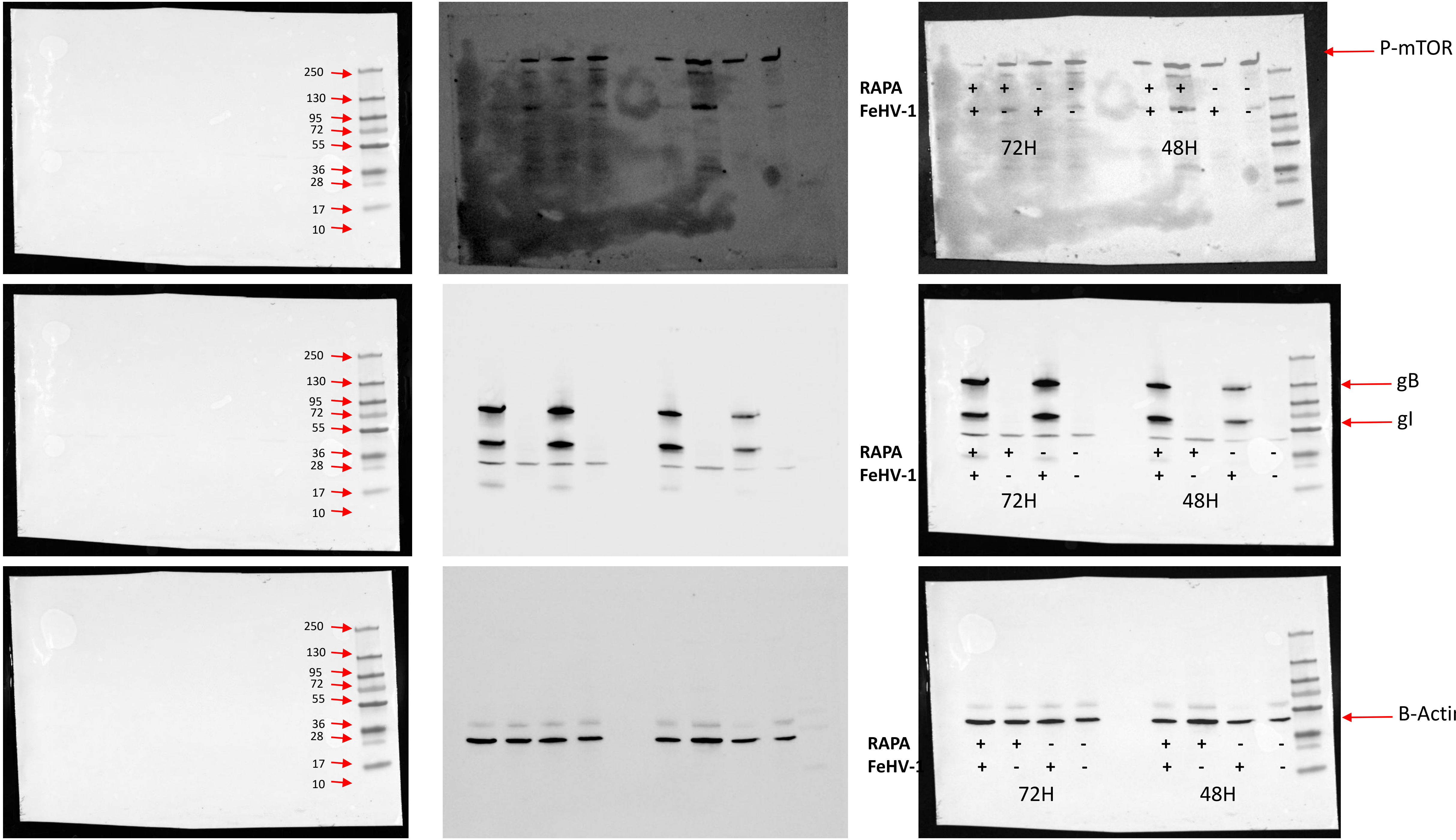

Fig 7

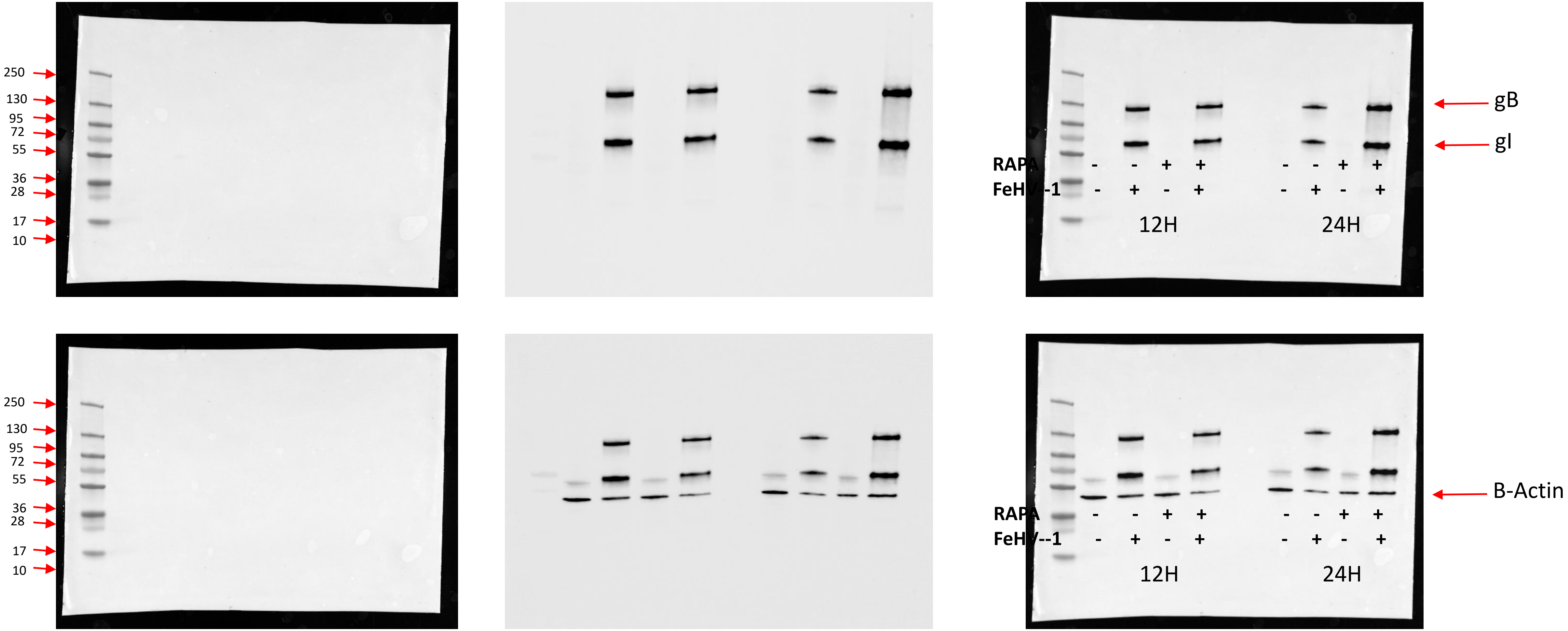
250
130
95
72
55
36
28
17
10
250
130
95
72
55
36
28
17
10
gB
gI
RAPA - - + + - - + +
FeHV-1 - + - + - + - +
12H
24H
B-Actin
RAPA - - + + - - + +
FeHV--1 - + - + - + - +
12H
24H

Fig 8

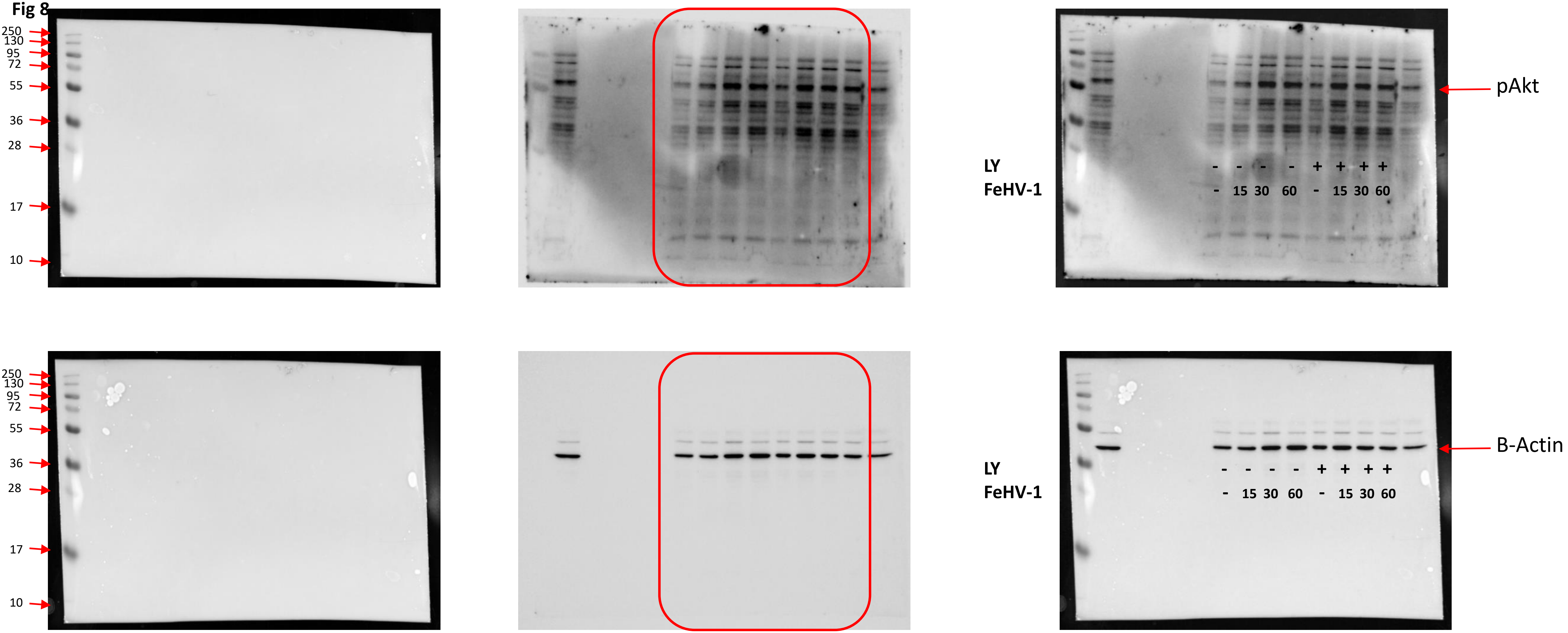
250
130
95
72
55
36
28
17
10
LY
FeHV-1
- - - - + + + +
- 15 30 60 - 15 30 60
pAkt
250
130
95
72
55
36
28
17
10
LY
FeHV-1
- - - - + + + +
- 15 30 60 - 15 30 60
B-Actin

Fig 9

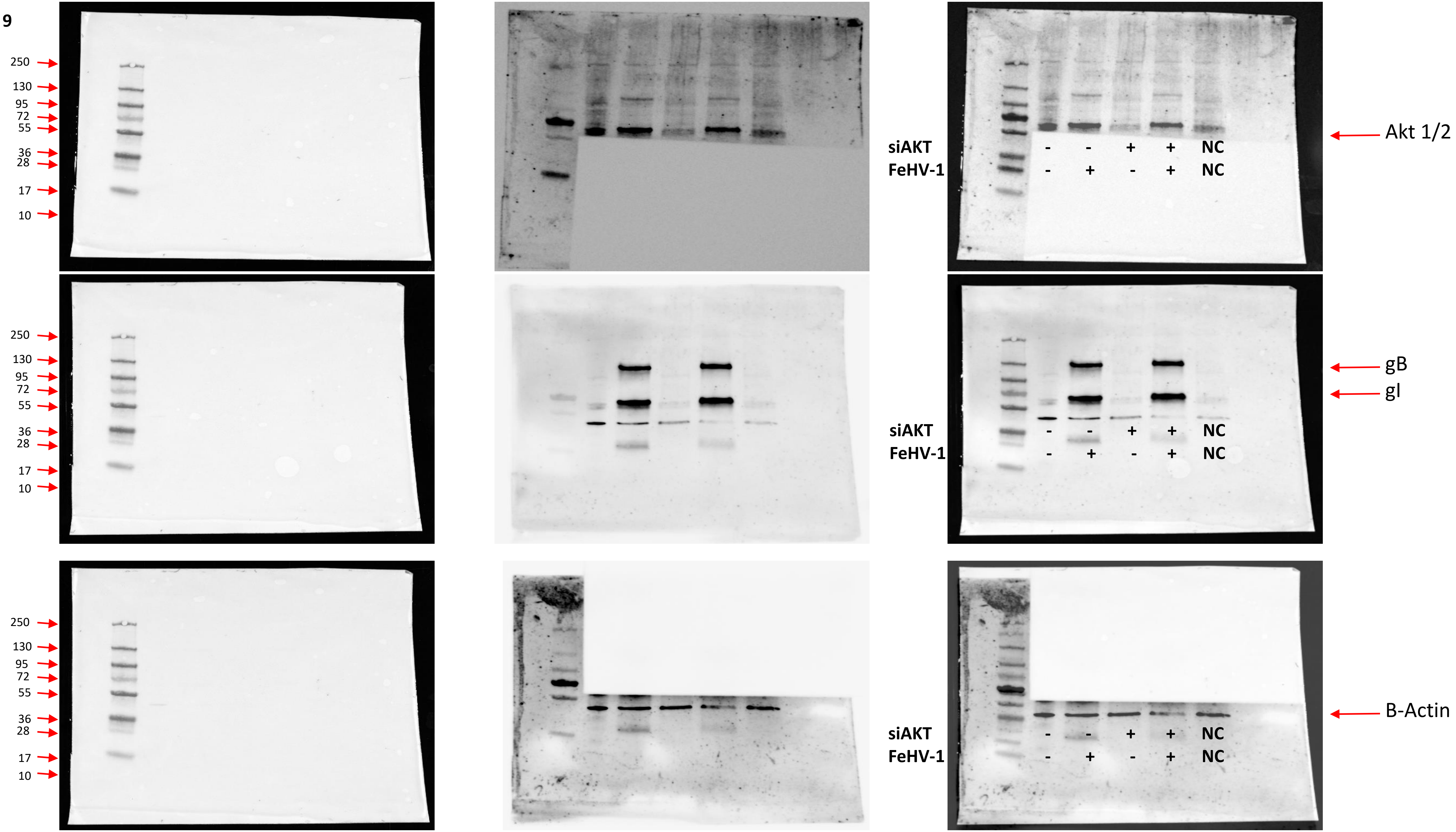

Fig 9

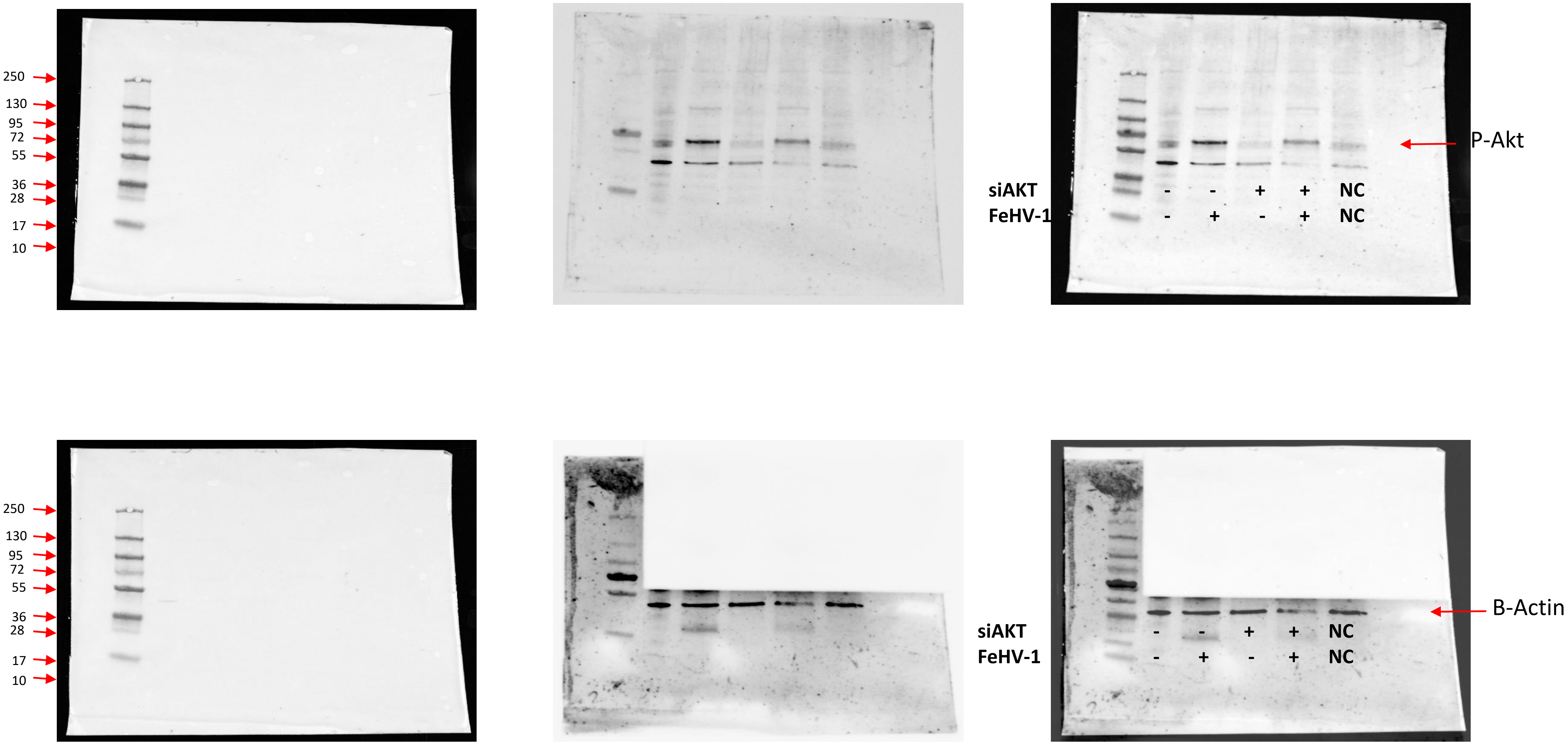
250
130
95
72
55
36
28
17
10
siAKT - - + + NC
FeHV-1 - + - + NC
P-Akt
250
130
95
72
55
36
28
17
10
siAKT - - + + NC
FeHV-1 - + - + NC
B-Actin
